# Multi-Omics Perspective Reveals the Different Patterns of Tumor Immune Microenvironment Based on Programmed Death Ligand 1 (PD-L1) Expression and Predictor of Responses to Immune Checkpoint Blockade across Pan-Cancer

**DOI:** 10.3390/ijms22105158

**Published:** 2021-05-13

**Authors:** Kaitang Huang, Meiling Hu, Jiayun Chen, Jinfen Wei, Jingxin Qin, Shudai Lin, Hongli Du

**Affiliations:** School of Biology and Biological Engineering, South China University of Technology, Guangzhou 510006, China; huangkaitang@foxmail.com (K.H.); humeiling@scut.edu.cn (M.H.); ninochenjiayun@foxmail.com (J.C.); 201810107408@mail.scut.edu.cn (J.W.); 201630845126@mail.scut.edu.cn (J.Q.)

**Keywords:** the Cancer Genome Atlas, immunotherapy, tumor immune microenvironment, programmed death ligand 1, tumor-infiltrating lymphocyte

## Abstract

Immune checkpoint inhibitor (ICI) therapies have shown great promise in cancer treatment. However, the intra-heterogeneity is a major barrier to reasonably classifying the potential benefited patients. Comprehensive heterogeneity analysis is needed to solve these clinical issues. In this study, the samples from pan-cancer and independent breast cancer datasets were divided into four tumor immune microenvironment (TIME) subtypes based on tumor programmed death ligand 1 (PD-L1) expression level and tumor-infiltrating lymphocyte (TIL) state. As the combination of the TIL Z score and PD-L1 expression showed superior prediction of response to ICI in multiple data sets compared to other methods, we used the TIL Z score and PD-L1 to classify samples. Therefore, samples were divided by combined TIL Z score and PD-L1 to identify four TIME subtypes, including type I (3.24%), type II (43.24%), type III (6.76%), and type IV (46.76%). Type I was associated with favorable prognosis with more T and DC cells, while type III had the poorest condition and composed a higher level of activated mast cells. Furthermore, TIME subtypes exhibited a distinct genetic and transcriptional feature: type III was observed to have the highest mutation rate (77.92%), while co-mutations patterns were characteristic in type I, and the PD-L1 positive subgroup showed higher carbohydrates, lipids, and xenobiotics metabolism compared to others. Overall, we developed a robust method to classify TIME and analyze the divergence of prognosis, immune cell composition, genomics, and transcriptomics patterns among TIME subtypes, which potentially provides insight for classification of TIME and a referrable theoretical basis for the screening benefited groups in the ICI immunotherapy.

## 1. Introduction

For the past few years, clinical results revealed that immune checkpoint inhibitor (ICI) treatment, such as programmed death-1 (PD-1) and its ligand 1 (PD-L1) checkpoint blockade, have shown an exhilaratingly long-term effect in a variety of cancer patients and have become a research focus in current tumor immunotherapy [1,2,3]. However, it has been reported that a number of patients showed a low response rate or treatment resistance against the anti-PD-1/PD-L1 checkpoint blockade [4,5,6]. Thus, it is significant to categorize patients into appropriate subpopulation, based on their cellular and molecular characteristics, to elucidate an inner mechanism, resulting in divergence of multi-omics patterns, and to ultimately provide clinical guidance on choosing corresponding treatment strategies for stratifying patients.

The various classifications of population-responding ICIs are mainly attributed to tumor microenvironments (TMEs), especially the composition and quantities of tumor-infiltrating lymphocytes (TILs), as well as numerous factors that independently predict clinical response to ICIs, including PD-L1 expression, tumor mutation burden (TMB), neo-antigen genotype, immune cell exhaustion, and disordered expression levels of cytokines [6,7,8,9,10]. It has been reported that the TIL status in the tumor immune microenvironment (TIME) is positively related to good clinical prognosis and could better predict the response to anti-PD-1/PD-L1 therapies [11,12,13,14]. Considering the inhibitory effect of cancer cells on the function of effector lymphocytes in TIME via immunological checkpoints, such as PD-L1, it is more comprehensive and precise to stratify TIME into different types by combining the two indicators above. Owing to the divergence of TIL status and PD-L1 expression, the immunologic effects of different TIME subtypes can be various, and thus, the corresponding immunotherapeutic strategies can be different. Recent research has described four different subtypes of TIME based on the positive or negative status of TIL and PD-L1 expression, including type I (PD-L1+/TIL+: adaptive immune resistance), type II (PD-L1−/TIL−: immunological ignorance), type III (PD-L1+/TIL−: intrinsic PD-L1 induction), and type IV (PD-L1−/TIL+: Other suppressors) [15,16,17], which may serve as a more systematic biomarker to stratify patients in clinical use of immunotherapy [18,19].

However, there are several issues that need to be addressed. First, most of these studies generally focused on one specific cancer type and classified samples into four subtypes to investigate their molecular characteristics without analyzing the multi-omics discrepancy of four subtypes in pan-cancer [16,20,21]. Second, they merely qualified the PD-L1 expression on the membrane surfaces of tumor cells by immunohistochemistry (IHC) [15,16,17,18,19,20]. However, several studies have reported that tumor cells are able to release a vast of exosomes, containing majority PD-L1, to suppress antitumor immunity rather than merely present PD-L1 on their cell surfaces [22,23]. This discovery may explain the discrepancy of PD-L1 expression between the transcriptomic level and proteomic level and reminds us that exclusive detection of expression of PD-L1 presenting on the membrane surface may have certain limitations. Third, they only evaluated the TIL status according to the CD8+T cell, which was the uppermost effector lymphocyte in TIME, without analyzing other kinds of functional lymphocyte impacts [15,19,20,21,24,25,26,27]. In most big cohort studies of immune-related cancer, researchers only used the expression levels of CD8+ T cell-related genes, such as CD8A or CD8B, to characterize TIL [15,24,25,26,27]. Additionally, they classified different patients into PD-L1 or TIL positive/negative subgroups without illustrating how threshold criteria were set, which was not reasonable for classification or further analysis [15,19,20,21,24,25,26,27]. Thus, the more precise indicator of TIL status, which reflects the interaction among various leukocytes in TIME, needs to be further studied.

In this study, we constructed a new method for classifying TIL states, which are an advanced predictor of responses to ICI. We then stratified patients into four TIME subtypes of 8634 samples overall across 33 cancer types from The Cancer Genome Atlas (TCGA) database, with more optimized classification methods. We analyzed the similarities and differences of distribution of 8 immune cell types in each subtype: T cells, B cells, macrophages, dendritic cells, natural killer cells, mast cells, neutrophils, and eosinophils. We also performed difference analysis of the genomic and transcriptomic level among four subtypes in order to elucidate the mechanism of TIME divergence. Hazard analysis was conducted to identify the impacts of several factors, including our classification patterns on survival statuses. Furthermore, we used 3069 breast cancer patients from the Gene Expression Omnibus (GEO) database for a similar classification study to verify the availability of analysis methods for widespread use. We believe that this stratification of cancer patients sheds light on new approaches to rationally apply the optimal cancer immunotherapeutic strategies for the four different TIME subtypes.

## 2. Results

### 2.1. Prognostic Significance of TIL Z Score/PD-L1 to ICI Response Prediction and Stratification of Four TIME Subtypes across Pan-Cancer Types

**Figure 1:**
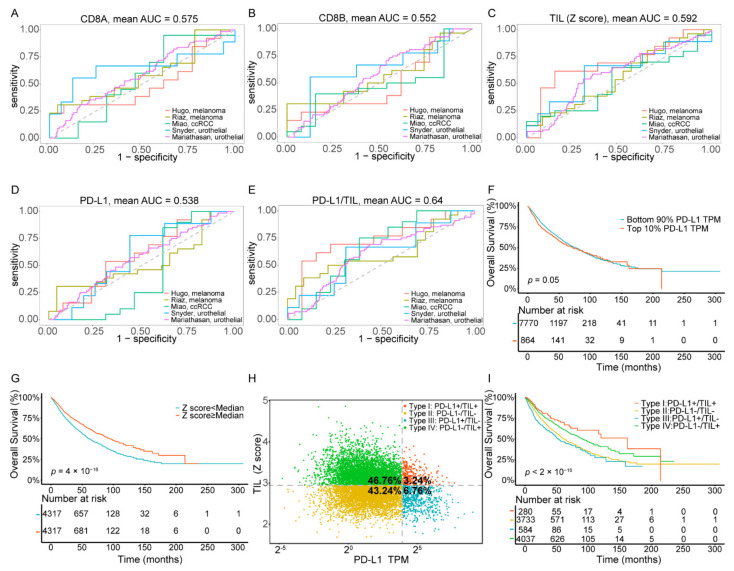
Combination of the TIL Z score and PD-L1 predicts clinical response to ICI immunotherapy and the stratification of four TIME subtypes across pan-cancer types. (**A**–**E**) ROC curves for the performance of CD8A, CD8B, the TIL Z score, PD-L1, and the combined TIL Z score with PD-L1 for predicting anti-PD-1 immunotherapy response in patients who received ICI therapy. (**F**) Kaplan–Meier survival curves of patients based on PD-L1 expression. (**G**) Kaplan–Meier survival curves of patients based on the TIL score. (**H**), The proportions of patients in type I, type II, type III, and type IV. (**I**) Kaplan–Meier survival curves of patients in type I, type II, type III, and type IV. Abbreviations: TIL: tumor-infiltrating lymphocyte, ICI: immune checkpoint inhibitors, TIME: tumor immune microenvironment.

Five published datasets [28,29,30,31,32] on PD-L1/PD-1 blockade immunotherapy, including pre-treatment transcriptome information and post-treatment clinical response data, were downloaded to evaluate and compare the performance of the TIL Z score and other common indicators, based on CD8A or CD8B expression, in predicting clinical response to ICI. We used the receiver operating characteristic (ROC) curve to measure the true-positive rates against the false-positive rates at various thresholds of the TIL Z score and CD8A and CD8B expression (Figure 1A–C). The results showed that the predictive power of the TIL Z score (AUC = 0.592) was higher than that of CD8A (AUC = 0.575) and CD8B (AUC = 0.552), which suggested that the TIL Z score had a strong robustness to ICI response prediction and was sufficient to characterize TIL. As PD-L1 was also related to ICI response, we assessed the AUC of PD-L1, and the result indicated that the predictive power of PD-L1 (AUC = 0.53) was lower than the TIL Z score (Figure 1D). We then combined PD-L1 expression and the TIL Z score to evaluate their performances. As Figure 1E shows, the combination of PD-L1 and the TIL Z score had a higher AUC (0.64) than others (0.53~0.59), which suggests that this combined index exhibits strong robustness to ICI response prediction (Table 1).

We analyzed 8634 tumor samples of 33 cancer types from the TCGA dataset using PD-L1 mRNA expression and the TIL Z-score to classify samples. The value distribution of PD-L1 expression varied according to the cancer types (ranging from 0.03 to 521.31, Figure S1A, Table S2), which reminded us that there may not be one universal definition of positive or negative PD-L1 expression for each cancer type. Thus, we defined PD-L1 subgroups by percentile rather than establishing a definitive cut-off value for PD-L1 expression. The cut-points chosen to define the PD-L1 positive subgroup were the top 10%, 20%, 30%, 40%, and 50% in each independent experiment. We then performed Kaplan–Meier survival analysis on each positive vs. negative PD-L1 group (Figure 1F, Figure S1B). Since patients had the most significant difference in overall survival state (Figure 1F) when the cut-point was set at the top 10%, this threshold was selected to determine the PD-L1 positive subgroup for further analysis.

Analogously, since the distribution of TIL in pan-cancer varied (ranging from 1.69 to 4.86, Figure S1C, Table S2), we classified TIL subgroups by percentile, and the cut-points chosen to define TIL positive group were the same as PD-L1. Our result of Kaplan–Meier survival analysis with a log-rank test showed a significant difference in positive vs. negative TIL groups (Figure 1G, Figure S1D). Here, we selected the top 50% of patients who exhibited the most significant difference in the overall survival state (*p* value = 4 × 10^−16^) to determine the TIL positive subgroup for further analysis. Particularly, our results of correlation analysis revealed a weak relationship (Spearman correlation, *p* value < 2.2× 10^−16^, R = −0.159) between TIL status and PD-L1 expression (Figure S1E), which indicated that the two indicators were mutually independent.

As the classification PD-L1 and TIL Z score showed prognostic significance in overall survival of cancer patients, respectively, we further intended to investigate the difference between subtypes in response to ICI treatment. We grouped ICI immunotherapy samples into four TIME subtypes by combining these two predictive indicators, and the result showed that the response rate was higher in type I (40%) and lower in types II and III (28.73%, 29.41%), which indicated that type I samples exhibited a more favorable response rate and may benefit from ICB immunotherapy (Figure S1F, Table S1).

We also grouped all TCGA cancer samples into four TIME subtypes by combining these two predictive indicators (Figure 1H). Among all patients, only 3.24% of the samples were classified as type I (PD-L1+/TIL+), while the proportions of type II (PD-L1−/TIL−), type III (PD-L1+/TIL−), and type IV (PD-L1−/TIL+) were 43.24%, 6.76%, and 46.76%, respectively. Additionally, these proportions were comparable to those reported previously (13.44~54%, 15.4~43.4%, 1~26.20%, 15.4~54.79%, respectively) [14,15,16,17]. The clinical, pathological, cellular, and molecular characteristics of overall cancer cases, according to TIME subtypes, are summarized in Table 2. Kaplan–Meier survival analysis of these four subgroups (Figure 1I) showed that the overall survival of patients within type I was significantly the most favorable, while the patients within type III showed the poorest prognostic condition. Notably, the TIL positive groups (type I and IV) had better survival outcomes than the TIL negative groups (type II and III), which revealed an association between TIL status and increased survival (*p* value < 2× 10^−16^).

Additionally, the proportion of four TIME types was calculated for a specific cancer type (Figure S1G and Table S2), occurring in different tissues, to display distribution of four subgroups. Notably, thymoma (THYM) was revealed to harbor the highest proportion of type I (55.26%) compared to other cancer types, indicating that tumors derived from lymphoproliferative organs are prone to form a lymphocyte-enriched immune microenvironment. Comparatively, the uterine carcinosarcoma (UCS) was majorly composed of type II (88.24%), while lung squamous cell carcinoma (LUSC) and liver hepatocellular carcinoma (LIHC) processed the highest proportion of type III (31.78%) and type IV (84.13%), respectively. In general, we stratified patients into four subtypes based on their PD-L1 expression and TIL status and proclaimed the distribution divergence of subgroups across pan-cancer. Our results support the hypothesis that combining these two indicators could better predict the prognostic status and the potential response levels of immunotherapy.

### 2.2. The Composition and Abundance of Lymphocyte among Four Subtypes

**Figure 2:**
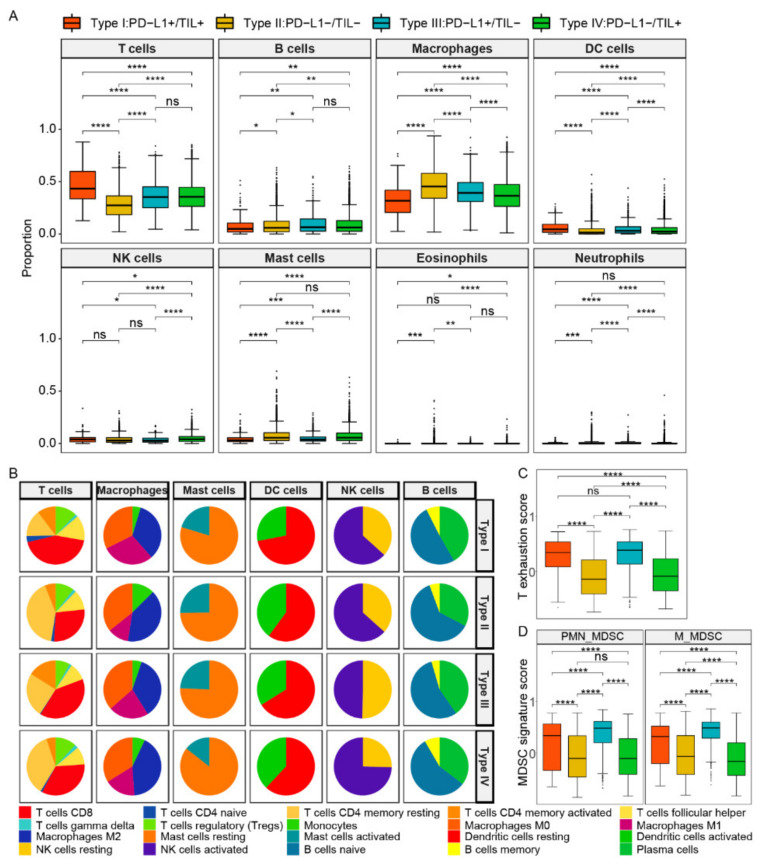
The composition and abundance of immune cells among four TIME subtypes. (**A**) The abundance difference among eight types of immune cells within four subtypes. (**B**) The abundance difference of six main subclass immune cells in each subtype. (**C**) The T cell exhaustion score between four subtypes. (**D**) The MDSC signature score between four subtypes. Abbreviations: M_MDSCs: monocytic MDSCs, PMN_MDSCs: polymorphonuclear MDSCs. ****, *p* < 0.0001; ***, *p* < 0.001; **, *p* < 0.01; *, *p* < 0.05.

Considering the differences of types and abundances of various immune cells would reflect the TIME, and studying the types and content of immune cells in TIME is of a great significance to further reveal immunization surveillance and malignant progression. We used the CIBERSORT tool to classify and estimate the level of immune cell infiltration by the deconvolution algorithm. Here, we divided 22 immune cells into 8 categories: T cells, B cells, macrophages, dendritic cells, natural killer cells, mast cells, neutrophils, and eosinophils. The abundance difference among 8 types of immune cells within four subtypes is shown in Figure 2A and Table S3. Compared to other subtypes, type I (PD-L1+/TIL+) contained a high level of T lymphocytes and DC cells but the lowest proportion of macrophages and mast cells (*p* values < 0.0001, respectively). In contrast, type II (PD-L1−/TIL−) had the lowest infiltrative levels of T lymphocytes and DCs in four subtypes, whereas its macrophage and mast cells were higher than other subtypes (*p* value < 0.0001, respectively). The infiltration level of NK cells in type IV (PD-L1−/TIL+) was the highest among four subtypes; however, its infiltration level of T lymphocytes was lower than that of the type I subtype (*p* value < 0.0001).

We further analyzed the details of immune cells in six main categories within four TIME types (Figure 2B, Table S4). In the T cells category, CD8+ T cells were mainly involved in killing tumor cells, as immune effector activity partially reflected by its content. Type I contained the most CD8+ T cells (43.56%) compared to other subtypes, while type III and type IV were closed to each other (39.43% and 33.81%, respectively), and type II was composed of the least CD8+ T cells (27.44%). The result revealed that better survival of patients may be related to the increased number of CD8 + T cells. The infiltration level of CD4+T memory resting cells in type I (15.03%) and type III (24.86%) were both less than that of type II (41.49%) and type IV (36.15%), yet the infiltration level of CD4+ memory T activated cells in type III was the highest (16.04%). T cells regulatory (Tregs) mainly play a role in suppressing immune cell effects, and their infiltration levels of type I, type II, and type IV were 12.11%, 10.53%, and 11.82%, respectively, which were higher than that of type III (8.05%), but the difference was not obvious. We supposed that Tregs content was not the main contributor to the difference in immune infiltration level.

As for other types of immune cells, the infiltration level of macrophages M2 was similar in type I, type II, and type III, which were 33.88%, 39.88%, and 35.87%, respectively, while that of type IV (45.42%) was higher than the other three types. In addition, the infiltration level of mast cells activated a range from 14.45% to 25.29% in four subtypes, while the TIL positive subgroups (type I/IV) were less than the other two types. The infiltrating level of DC activated cells of type I was the least, while that of type II, type III, and type IV were 39.91%, 33.92%, and 38.02%, respectively. Additionally, the content of NK activated cells in type IV exceeded the other three subtypes, ranging from 49.76% to 74.56%. Notably, the infiltration levels of the subpopulation of B cells were closer in composition among the four subtypes. Additionally, as myeloid-derived suppressor cells (MDSCs) infiltration and the T cell exhaustion state were revealed to be associated with immunosuppression, we further explored the comparison of proportion of MDSCs and the state of T cell exhaustion between the four subtypes. It was observed that the T cell exhaustion score was higher in PD-L1 positive groups (subtype I and subtype III), but there were no significant differences between subtype I and subtype III (Figure 2C). The results showed that the scores of both polymorphonuclear MDSCs (PMN- MDSCs) and monocytic MDSCs (M- MDSCs) were the highest in subtype III (PD-L1+/TIL−), and higher in PD-L1 positive groups compared to negative groups, and higher in TIL negative groups compared to positive groups (Figure 2D).

In general, the TIL positive subgroups that acquired good survival outcomes contained a high proportion of key immune cells, including activated CD8+T cells and NK cells. We speculated that the immunophenotype difference in four subtypes may be due to the abundance difference of these divergent cells.

### 2.3. Genomics Pattern Discrepancy in Four TIME Subtypes

**Figure 3:**
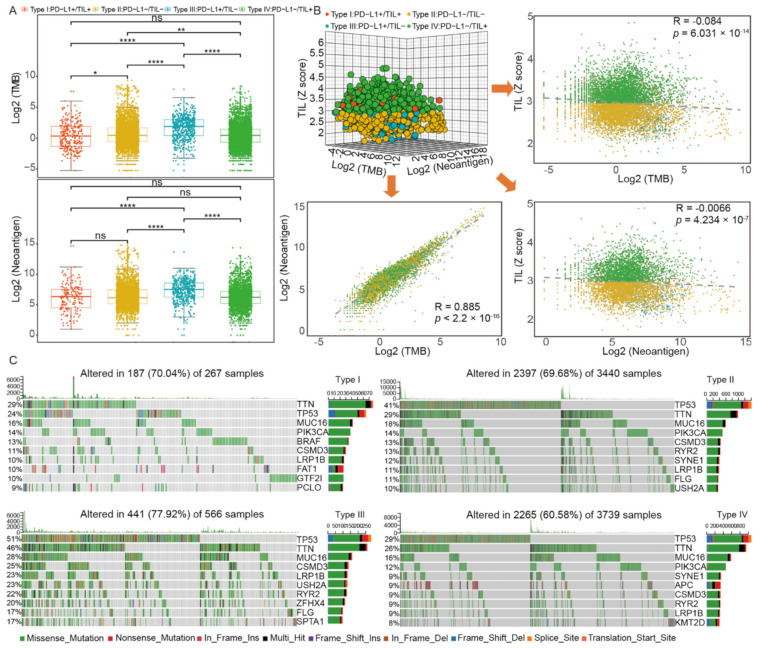
The genomics pattern discrepancy in four TIME subtypes. (**A**) The distribution of TMB and neoantigen among four subtypes; (**B**) correlation analysis among TIL, TMB, and neoantigen; (**C**) the alteration landscape of somatic variants across four subtypes. ****, *p* < 0.0001; ***, *p* < 0.001; **, *p* < 0.01; *, *p* < 0.05.

Here, we investigated the discrepancy of TMB and neoantigen among four subtypes (Figure 3A, Table S5) and we found that type III had a remarkable high somatic mutation burden and neoantigen compared to others (*p* value < 0.0001). As for type I, type II, and type IV, there were no significant differences of neoantigen, as well as type I and type IV of TMB. We also constructed a 3-dimensional dot plot base on TIL, TMB, and neoantigen and performed linear regression analysis between every two factors (Figure 3B). Notably, a statistically significant correlation between the TMB and the neoantigens number was found (Spearman correlation, R = 0.885, *p* value < 2.2 × 10^−16^, Figure 3B). However, there was no significant correlation between TMB and TIL (Spearman correlation, R = −0.084, *p* = 6.031 × 10^−14^, Figure 3B) or neoantigen and TIL (Spearman correlation, R = −0.066, *p* = 4.234 × 10^−7^, Figure 3B). A correlation between PD-L1 expression and TMB or neoantigen was not found either (Spearman correlation, R = 0.099, *p* value < 2.2 × 10^−16^ and R = 0.151, *p* value < 2.2 × 10^−16^, respectively) (Figure S2A, Figure 2B).

We sought to investigate the alteration landscape of somatic variants across four subtypes and the specific tumor mutation spectrum, the frequencies of which varied in the top 10 mutant genes. Generally, we found that the patients of type III had the highest altered rate (77.92%) among four subtypes. As shown in Figure 3C and Table S6, five gene mutations were found in all four subtypes: *TTN* (29%, 29%, 46%, and 26%, respectively), *TP53* (24%, 41%, 51%, and 29%, respectively), *LRP1B* (10%, 11%, 23%, and 9%, respectively), *MUC16* (16%, 18%, 28%, and 16%, respectively), and *CSMD3* (11%, 13%, 25%, and 9%, respectively). Specifically, compared to others, tumors of type III acquired the highest mutation rate of these five genes. We then investigated the unique mutated genes of each subtype: *BRAF* (13%), *FAT1* (10%), *GTF2I* (10%), and *PCLO* (9%) in type I, *ZFHX4* (20%) and *SPTA1* (17%) in type III, *APC* (9%) and *KMT2D* (9%) in type IV, and no unique characteristics of type II mutated genes. We further investigated the relationship between TIL and gene mutation. The result indicated that there was a statistically significant difference of TIL status between the *TP53* mutation subgroup and the wild-type subgroup (*p* value < 0.0001, Figure S2C) and the proportion of patients who were TIL positive in the wild-type subgroup was higher than that of the mutation subgroup (Figure S2D).

Considering that many mutated genes were co-occurring or displayed strong exclusiveness, we then explored the potential different somatic interactions among four subtypes to expound their mutation pattern (Figure S2E). The interaction of these genes with oncogenes suggests a close relationship to cancer occurrence and development. In type I, specific interaction patterns were found: *GTF2I* and *BRAF* mutations were both significantly mutually exclusive to other gene mutations (*p* value < 0.01, respectively), while the other mutations co-occurred more obviously. However, in type II, type III, and type IV, most of the gene mutations were significantly co-occurring (*p* value < 0.01), except for *TP53* with *SYNE1* in type II and *PIK3CA* with *TP53* in type IV. We further evaluated and identified oncogenes in each subtype (Figure S2F, Table S7). Type IV owned the most oncogenes (16 in total) compared to the other three subtypes, and the most common oncogene *KRAS* mutation appeared across all four subtypes. Of these oncogenes, three of them (*GTF2I*, *BRAF*, and *PIK3CA*) had relatively higher mutated frequencies in type I compared to the other three subtypes. In addition, the *BRAF* mutation subgroup had a higher proportion of patients who were TIL positive (*p* value < 0.0001, Figure S2G, Figure S2H). However, the difference of TIL levels between the HRAS mutation subgroups and wild-type subgroup was not found, though HRAS mutation was uniquely identified in the TIL positive subgroup (type I/IV) (*p* value = 0.78, Figure S2I). Furthermore, different PD-L1 expression between the IDH-1 mutation subgroup and wild-type subgroup was statistically significant (*p* value < 0.0001, Figure S2J). In conclusion, the specific somatic mutation spectrum of each subtype could help us accurately classify patients into such subgroups.

### 2.4. Transcriptomics Pattern Discrepancy in Four TIME Subtypes

**Figure 4:**
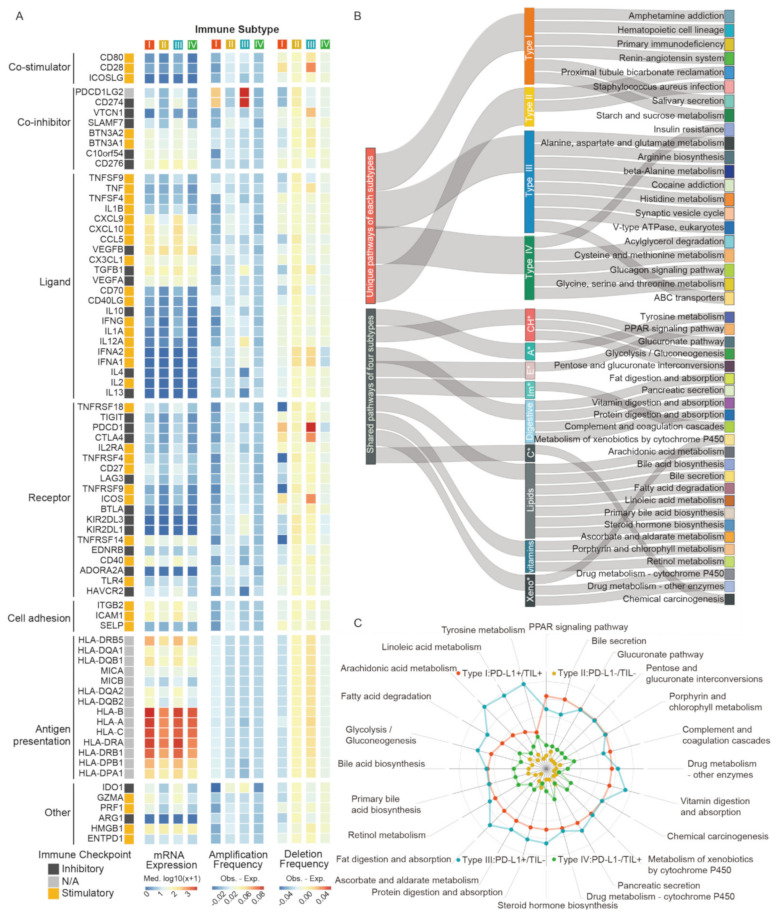
The transcriptomic pattern discrepancy in four TIME subtypes. (**A**) The immunomodulators gene expression and copy number variation for each subtype. (**B**) The shared and unique pathway features for each subtype. (**C**) The distinct difference weight score of pathways in each group. Abbreviations: CH*: carbohydrates, A*: Amino acid, E*: Endocrine, Im*: Immune, C*: Cancer, Xeno*: Xenobiotics.

Understanding the divergence of immunomodulators (IM) expression and state is critical to descript transcriptomics patterns of each subtype. We thus examined the IM gene expression, as well as copy number variation (CNV), including amplification and deletion (Figure 4A, Table S8). In general, the gene expression differences of IMs across immune subtypes were not significant. Thereinto, PD-L1 positive subgroups (type I/III) presented similar states in co-inhibitor, ligand, receptor, and other modulators, as their gene expression levels were largely higher than PD-L1 negative groups (type II/III). For copy number alterations, type I generally showed low frequency amplification and deletion of IM genes, except for IM genes *PDCD1LG2* and *CD274* (PD-L1), which amplified a higher frequency, and noticeably, these genes had the highest frequencies in type III. Additionally, *CD28*, *VTCN1*, *PDCD1*, *CTLA4*, and *ICOS* had higher frequency deletion in type III as well. We found that the PD-L1 expression level in *PDCD1LG2* and *CD274* copy number amplification subgroups were higher than that of non-amplification subgroups (*p* value < 0.0001, Figure S3A,B, respectively), but *PDCD1* or *CTLA4* subgroups suggested opposite conclusions (*p* value < 0.01 & < 0.0001, Figure S3C,D, respectively). In conclusion, these marked divergences in IM genes clarified the perspective of PD-L1 subgroups referring molecular patterns discrepancy, which may be reflective of the immunomodulator state of the TIME in patients.

To reveal the key deregulated pathways occurring in each subtype, we analyzed different gene expression and calculated gene scores based on log fold changes values by comparing samples within one subtype with the other three integrated samples. Magnitude of pathway dysregulation was calculated by gene scores and assigning scores, based on the enrichment pathways of different expressed genes (DEGs) from The Kyoto Encyclopedia of Genes and Genomes (KEGG). As shown in the result, four TIME subtypes exhibited common signatures but maintained some unique features of their own (Figure 4B). Type I exhibited six unique pathways, including amphetamine addiction, hematopoietic cell lineage, primary immunodeficiency, renin-angiotensin system, salivary secretion, starch, and sucrose metabolism. Proximal tubule bicarbonate reclamation and staphylococcus aureus infection were the only unique pathways activated in type II. Notably, the most common pathways showed in type III were metabolic-related processes, such as alanine, aspartate, and glutamate metabolism, arginine biosynthesis, and ABC transporters. The specific pathway terms in type IV were also different, such as the glucagon signaling pathway and cysteine and methionine metabolism. We deemed that dysregulation of unique pathways in each subtype suggested different TIME signatures and potential differential sensitivity, providing the fundamentals of theoretical mechanism research for therapeutic intervention. We also determined the distinct difference weight scores of pathways in each subtype, which indicate enrichment degree and differential status of DEGs (Figure 4C, Table S9). With few exceptions (e.g., immune system, carcinogenic process), there was significant enrichment in metabolic genes that were frequently shared across all subtypes, but to a different degree. Overall, type I and type III (PD-L1+) harbored higher pathway scores than the other two types. Specifically, type III exhibited the highest score of pathways, except for PPAR signaling, bile secretion, and complement and coagulation cascades, while some were consistent with type I. Compared to type II and type IV (PD-L1−), these pathways in type IV changed more dramatically.

We then analyzed the expression distributions of cytokines and cytotoxic-related genes in each subtype found that the gene expression of immuno-activation cytokines (*IFNG*, *TNF*, *IL12A*, and *IL12B*), immuno-suppressive cytokines (*TGFB1*, *IL6*, and *IL10*), and cytolytic factors (*GZMB* and *PRF1*) were largely higher in type I and type III (PD-L1+) than the other two PD-L1 negative subtypes (Figure S3E, Table S10), which may indicate that PDL1 expression is involved in regulating immune balance. Moreover, there were also differences in cytokines observed among types I and III or typed II and IV. *TGFB1*, *IL6*, and *IL10* were higher in TIL negative groups compared to TIL positive groups, which indicated the potential immunosuppressive effects brought by these cytokines. However, *IFNG*, *TNF*, and *IL12A* were also higher in type III compared to type I and higher in type II compared to type IV, suggesting the complexity of the immune microenvironment (Figure S3E, Table S10). Moreover, it emerged that the tumor vasculature itself constituted an important barrier to T cells. We analyzed the association between angiogenesis-related growth factors, as well as their receptors with TIL subtypes. We found that expression of *EDN1*, *EDNRA*, *VEGFB*, *KDR*, and *FLT1* were higher in TIL negative groups; to be specific, they were higher in type III compared to type I and higher in type II compared to type IV (Figure S3F,G). Additionally, the correlation analysis showed the gene expression of growth factors and receptors were negatively correlated with the TIL score; *EDNRA* especially exhibited a higher negative correlation coefficient with the TIL score (Figure S3H). These results further suggested the adverse effects of tumor vasculature disorder on TIL.

### 2.5. Hazard Analysis for Multiple Omics Factors across Four TIME Subtypes

**Figure 5:**
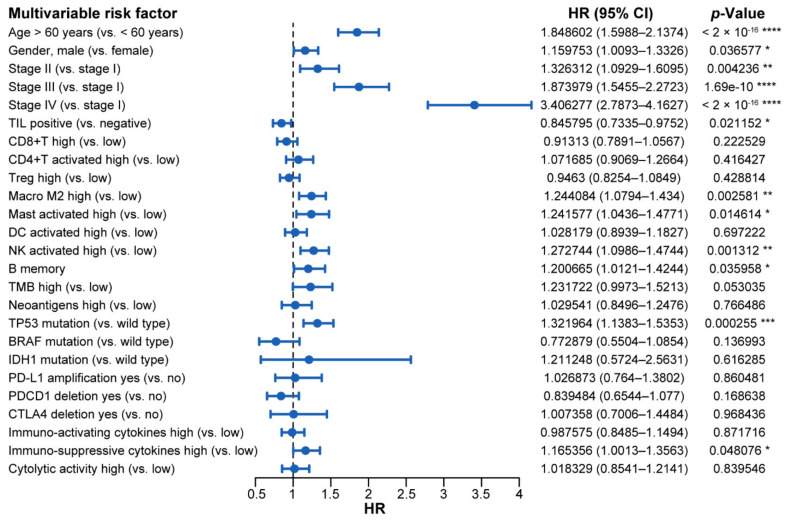
The forest plot of prognostic values for multivariable cox proportional hazard regression models. Abbreviations: HR: hazard ratio; CI: confidence interval; TIL: tumor infiltrating lymphocyte; TMB: tumor mutation burden. ****, *p* < 0.0001; ***, *p* < 0.001; **, *p* < 0.01; *, *p* < 0.05.

Significant variables (*p* value < 0.05) of the univariate analysis were into entered a multivariate Cox model. In the model, we examined several factors, including age, gender, tumor stage, TIL status (overall and specific cell types), TMB, neoantigen level, *TP53*, *BRAF*, and *IDH1* mutation state, copy number variation of *PD-L1*, *PDCD1*, and *CTLA4*, and immuno-activating/suppressive cytokines and cytolytic activity (Figure 5, Table 3). We found that positive TIL was associated with a good prognosis and higher overall survival (Hazard Ratio (HR): 0.846; 95% CI: 0.734–0.975; *p* value = 0.02). In contrast, high Macrophage M2 and activated mast cells were associated with significantly higher overall mortality and were not conducive to survival (HR: 1.244; 95% CI: 1.079–1.434; *p* value = 0.0026 and HR: 1.242; 95% CI: 1.044–1.477; *p* value = 0.015, respectively). Furthermore, an advanced tumor stage, such as stage IV (HR: 3.406; 95% CI: 2.787–4.163; *p* value < 2 × 10^−16^) and stage III (HR: 1.874; 95% CI: 1.546–2.272; *p* value = 1.69 × 10^−10^), a high level of immuno-suppressive cytokines (HR: 1.165; 95% CI: 1.001–1.356; *p* value = 0.048), and *TP53* mutation (HR: 1.322; 95% CI:1.138–1.535; *p* value = 0.000255) were all associated with poorer overall survival.

### 2.6. Validation in GEO Dataset

**Figure 6:**
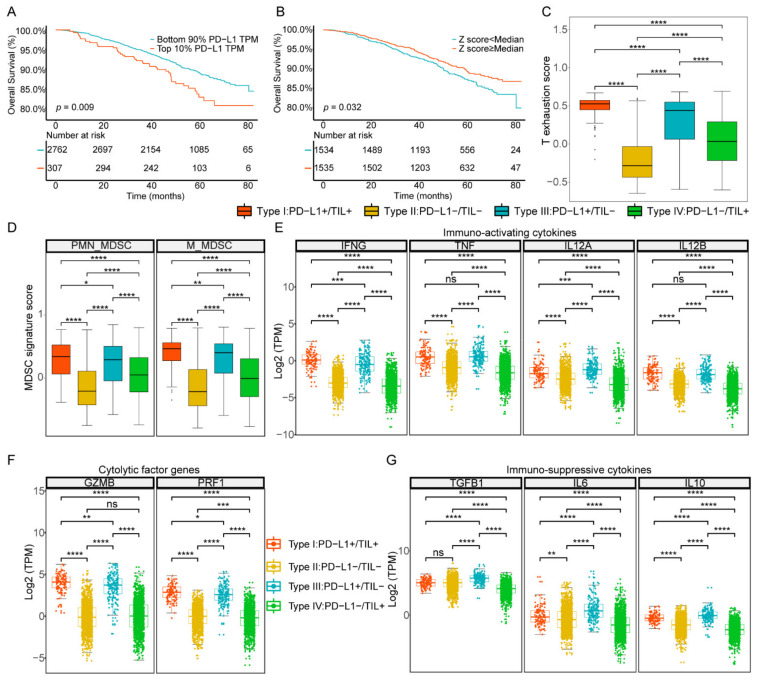
Stratification of four TIME subtypes in the GEO database. (**A**) Survival analysis of positive vs. negative PD-L1 groups. (**B**) Survival analysis of positive vs. negative TIL groups. (**C**) The T cell exhaustion score between four subtypes. (**D**) The MDSCs signature score between four subtypes. (**E**–**G**) The gene expression distributions of cytokines and cytolysis factors in each subtype. ****, *p* < 0.0001; ***, *p* < 0.001; **, *p* < 0.01; *, *p* < 0.05.

To further validate the widespread use of this classification method based on PD-L1 and TIL level, we performed similar analysis at a public mRNA expression dataset (GSE96058) containing sufficiently large numbers of breast cancer samples (*n* = 3069) deposited in GEO. As before, we set the intervals that define PD-L1 and TIL positive to multiple percentiles: top 10%, 20%, 30%, 40%, and 50%. We then performed the Kaplan–Meier survival analysis log-rank test and found that, when PD-L1 and TIL positive were in the top 10% (*p* value = 0.009) and top 50% (*p* value = 0.032), respectively, the difference of the overall survival curve was the most significant, which was consistent with the results of TCGA dataset analysis, indicating that the thresholds we took were appropriate (Figure 6A,B, Figure S4A,B). We further grouped the GEO samples into four TIME subtypes based on the combination of PD-L1 and TIL, as previously described. The difference of overall survival curve of the four subtypes was statistically significant (*p* value = 0.015), the prognosis condition of type III was poorest, and the survival outcomes of the TIL positive groups (type I and IV) were better than the TIL negative groups (type II and III), which were similar to the results of TCGA dataset analysis, but the prognosis condition of type I was not the most favorable, unlike the TCGA dataset analysis (Figure S5A). Among all patients in GEO validation, the proportions of type I, type II, type III, and type IV were 3.68%, 43.66%, 6.32%, and 46.34%, respectively, which was similar to the results of the TCGA cohort (Figure S5B).

As before, we used the CIBERSORT tool to classify and evaluate the infiltration level of immune cells. The abundance difference among eight types of immune cells within four subtypes was shown in Figure S5C and Table S11. Analogously, type I (PD-L1+/TIL+) contained the highest level of T lymphocytes and the lowest proportion of macrophages and mast cells (*p* values < 0.0001, respectively), and type II (PD-L1−/TIL−) had the lowest infiltrative levels of T lymphocytes and the highest level of macrophage and mast cells (*p* value < 0.0001, respectively), and the infiltration level of T lymphocytes of type IV was lower than that of the type I subtype (*p* value < 0.001). However, there was no significant difference in the abundance of DC cells among the four TIME subtypes.

The proportion of 20 immune cell types classified into 6 main cell types among the 4 TIME subtypes was shown in Figure S5D and Table S12. Type I contained the most CD8+ T cells (37.82%) compared to other subtypes, while type II was composed of the least CD8+ T cells (29.39%) and type III and type IV were closed to each other (34.83% and 31.91%, respectively). The infiltration level of CD4+T memory resting cells in type I (43.69%) and type III (48.32%) were both less than that of type II (57.34%) and type IV (55.23%). The infiltration levels of T cells regulatory (Tregs) among type I, type II, type III, and type IV were 8.44%, 5.88%, 7.42%, and 6.64%, respectively, the difference of which was not obvious. The infiltration level of macrophages M2 of type II (64.74%) and type IV (62.46%) were higher than type I (47.91%) and type III (52.55%). The infiltration levels of mast cells of type I (2.8%) and type IV (4.8%), which belong to TIL positive subgroups, were lower than type II (6.15%) and type III (7.92%). The infiltration levels of the B cells subpopulation were closer in composition among the four subtypes. As above, the proportions of cell types among the four types were similar to those in the results of the TCGA dataset. We also explored the comparison of proportion of MDSCs and the state of T cell exhaustion between four subtypes in the GEO dataset. It was observed that the T cell exhaustion score was higher in PD-L1 positive groups (subtype I and subtype III) but higher in subtype I compared to subtype III. The results showed that the scores of both PMN-MDSCs and M-MDSCs were higher in PD-L1 positive groups compared to negative groups and higher in TIL positive groups compared to negative groups (Figure 6C,D).

As in the TCGA dataset analysis, the expression levels of immuno-activation cytokines (*IFNG*, *TNF*, *IL12A*, and *IL12B*), immune-suppressive cytokines (*VEGFA*, *TGFB1*, *IL6*, and *IL10*) and cytolytic factors (*GZMB* and *PRF1*) were higher in the PD-L1 positive subtypes (type I and type III) than in the PD-L1 negative subtypes (type II and type IV) (Figure 6E–G, Table S13). Consistent with the TCGA results, we found that the expression of growth factors and their receptors were higher in TIL negative groups (Figure S5E,F). Additionally, the correlation analysis showed the gene expressions of growth factors and receptors were also negatively correlated with the TIL score (Figure S5G). In general, GEO dataset results showed a similar pattern to that of the TCGA dataset, no matter the classification of PD-L1 and TIL, the composition of immune cells, or the expression of transcriptome, indicating the reliability of our results and universality of the classification method.

## 3. Discussion

PD-L1, as an immune checkpoint, is generally upregulated in TIME and promotes immune escape of tumor cells [33,34]. As a main target of immunotherapy, PD-L1 immunoblockade therapy brings great benefits to many patients, but its clinical application still has certain limitations. For example, many studies have found that the PD-L1 expression state is not directly correlated to the response rate or immunotherapy prognosis in different cancer types [35,36]. In this study, using a large scale of TCGA pan-cancer datasets, we systematically investigated the distribution of PD-L1 expression and TIL status, examined their prognostic impacts, and stratified 8634 patients into four subtypes across 33 cancer types by combining these two factors. We also used the GEO breast cancer dataset to validate our findings and found analogous conclusions. Although a positive correlation between PD-L1 expression and CD8+T cells was reported by previous researches [37,38], our results showed that TIL status was independent of PD-L1 expression, which allowed a further reasonable classification. Overall survival analysis illustrated that patients in TIL+ groups (type I and type IV) had better prognostic outcomes than that in TIL− groups (type II and type III), which were consistent with the prognostic outcome of TIL alone. Type I has a higher survival rate than that of type IV, suggesting that the prognostic outcome of PD-L1+/TIL+ subtypes was better than that of PD-L1−/TIL+ results, which is inconsistent with some previous studies [20], since only CD8+ T cells were considered as TILs in their research. Notably, the lower proportion of PD-L1 positive subtypes (type I and III) that was revealed by our study may imply a relative low proportion of patients who would potentially benefit from PD-L1 immunosuppressor. In particular, the distribution of four subtypes varied among the 33 cancer types, which inspired us to consider that different immunotherapy strategies should be adopted for different cancer types, even different patients with the same kind of cancer, to achieve precise treatment effect [20].

The TIME is a bidirectional, dynamic, and intricate interaction network between tumor cells and non-malignant cells, including immune cells and stromal cells [11,39]. Among them, owing to the difference of types and abundance of various immune cells, the formation of different TIME types could guide the tumor occurrence, development, and even transfer patterns. Therefore, analyzing the type and abundance of immune cells in corresponding subtypes of TIME is of great significance for further revealing the molecular mechanism of tumorigenesis and malignant progression [40,41]. Our results show that CD8+T cells and DC cells in type I were richer than the other three subtypes. We believe that the higher CD8+T cell infiltration level may endow type I patients with higher immunity, since the cytolytic activity-related gene *GZMB* and *PRF1* expressions were also higher in type I, as shown in transcriptome analysis, thus giving a more promising prognostic effect. The proportion of T cells of type IV was lower than that of type I, while its content of NK-activated cells was higher than that of type I. We hypothesize that the tumor killing effect of type IV patients is more dependent on NK cells. The intrinsic mechanism of different subtypes in recruiting T cells and NK cells, particularly the presence of PD-L1, remains to be elucidated. T cell exhaustion state was higher in PD-L1 positive groups, which further suggest the strong association between PD-L1 signals and T cell exhaustion. Of immune cells that exert immunosuppressive effects, Treg cells were not responsible for differences in immune microenvironment, but TIL negative groups had higher rates of MDSCs compared to the positive subtypes, as well as the relatively high proportions of M2 macrophage. Therefore, we reasoned that MDSCs and M2 macrophage were important factors to prevent T cell infiltration, and the difference of immune microenvironment in different subtypes is mainly reflected by a relative abundance of CD8+ T cells, MDSCs, and M2 macrophage [42,43].

Previous research has reported that TMB and neoantigen were associated with better immunotherapy effect, but its predictive effect has a limited effect on certain cancers, such as non-small cell lung cancer and colorectal cancer [44,45]. Our results reveal a significant correlation between TMB and neoantigens, but the relationships between TIL and TMB or neoantigen were not found. Therefore, we expect that high TMB or neoantigen would not primarily lead to high levels of immune infiltration, which remind us that novel and robust factors predicting the immunotherapy effect for various cancer should be further discovered. We also investigated the mutation landscape of high frequency foreach subtypes. For high frequency mutated genes of specific subtype, gene BRAF in type I encodes a protein belonging to the RAF family of serine/threonine protein kinases, which have been identified in various cancers [46]. Some research has reported that BRAF V600E mutation would sustain IFN-γ inducible PD-L1 expression by coactivating STAT1 and increasing protein translation and is associated with high levels of PD-L1 expression [47,48,49,50], and the patients with BRAF mutations appeared to benefit from monotherapy with PD-L1 inhibitors, which is consistent with results of the present study, to some extent. APC gene in type IV encodes a tumor suppressor protein that acts as an antagonist of the Wnt signaling pathway, which was involved in other processes, including cell migration and adhesion, transcriptional activation, and apoptosis [51]. However, we did not retrieve any reports concerning the relationship between this gene mutation and PD-L1 expression. As for oncogenes for each subtype, we found that KRAS mutation was the most common oncogene, while some studies reported that PD-L1 expression was upregulated by KRAS G12D mutation and KRAS mutations could serve as a potential predictor of anti-PD-1/PD-L1 immunotherapy [52,53]. In general, gene mutation spectrums present genomics divergence among four subtypes, and, in the future, highly specific targeted drugs for different patients need to be used to maximize the therapeutic effect, and the combination of targeted therapy and immunotherapy will be a promising treatment.

The divergence of transcriptomic patterns between PD-L1 positive groups and PD-L1 negative groups demonstrated that difference of IM gene expression pattern might attribute to a PD-L1-related pathway, while this assumption needs further confirmation. In the unique pathway studies, the association of immune types with signaling pathways was investigated based on RNA expression data of DEGs. Type I was associated with hematopoietic cell lineage, which could be contributed to CD4+T cells, suggesting a fundamental role of TIL in hematopoiesis through the secretion of cytokines or interferon [54]. It is the arginine biosynthesis pathway that mainly draws our attention to type III, whose concentrations impact the metabolic fitness directly and T cells capacity, which are crucial for anti-tumor functionality in TIME, as previously reported [55]. Arginine biosynthesis is more active in the cancer cells of type III and indicate that lack of arginine, because of weak competition in immune cells, may lead to energy depletion and less TIL in local TIME. Combined with previous research, our results suggest that TIL is associated with multiple biological states, such as genesis of blood cells and synthesis and metabolism of amino acid in TIME. Other TIL-related factors need further confirmation. 

The common pathway analysis revealed distinguishing patterns of activity shared by four subtypes. Surprisingly, type I and type III exhibited higher scores in most shared metabolism pathways, suggesting that PD-L1 high expression is more likely associated with metabolic alternations in TIME. The previous study discovered an unexpected role for PD-L1 in regulating tumor cell metabolism in the D42m1-T3 mice model. Specifically, PD-L1 could enhance the glycolysis of tumor cells by association with some signaling proteins, such as mTOR [56]. Combined with our study, higher PD-L1 expression may affect certain energy metabolism in tumor cells and thus weaken the nutrient intake of immune cells due to competition in type I and type III, compared to type II and type IV. Xenobiotics was metabolized by cytochrome P450, which could be induced by aryl hydrocarbon receptor (AHR) activation [57]. Type I and type III exhibit a more active xenobiotics metabolism, while type II and type IV show less, indicating that xenobiotics metabolism may influence PD-L1 expression through AHR signaling in TIME [58]. More work is required to determine how PD-L1 signals and the accurate connection between PD-L1 and metabolic pathways or biological processes in tumor and immune cells. Moreover, malignant cells can deprive glucose in TIME, thus blocking effective anticancer immunity, as glucose is used by T cells, NK cells, macrophages, and DCs to support their effector functions [56,59]. Glycolysis was shown to regulate TIL on account of metabolic competition in the tumor microenvironment, which can blunt Ca2+ signaling, glycolytic capacity, and cytokine production of TILs because glucose consumption by tumors metabolically restricts T cells [59,60]. Our research shows that cancer cells in type III (PD-L1+/TIL−) had more active glycolysis, suggesting that glycolysis is vital to TIL and is affected by PD-L1 expression. In summary, PD-L1 positive subtype (type I/III) and PD-L1 negative subtype (type II/IV) harbor distinct alterations in cell metabolism pathways, while the TIL subtypes have minor differences, and it seems that there are more potential connections between PD-L1 and TIME metabolism. These results may catalyze a better understanding of the role of immune cells’ altered metabolism in anti-cancer ability and provide novel means to stratify patients based on metabolic features and immunological status. Moreover, gene expression of endothelial-related growth factors and receptors were lower in TIL positive groups, which suggest that endothelial tumors and disorganized vasculature establish the barrier preventing T cell infiltration into tumors [61].

Hazards analysis identified several reliable indicators for evaluation of clinical treatment effects, except for common factors present, such as age, gender, and tumor stage. Most of the multivariable prognostic factors, such as macrophages M2, activated mast cells, *TP53* mutation, and immuno-suppressive cytokines expression, are unfavorable for survival by promoting the occurrence and development of tumors. In particular, TIL is implied to reduce the risk of death and is considered a good prognostic factor in cancer patients. In addition, consideration of the combination of more factors may improve sensitivity or specificity of clinical diagnosis and treatment.

## 4. Materials and Methods

### 4.1. Data Collection and Preprocessing

Immunotherapy dataset: Pre-treatment transcriptome information and post-treatment clinical response data from the five datasets of previous studies [28,29,30,31,32], whose patients received anti-PD-1 or anti-PD-L1 immunotherapy and were downloaded to evaluate the power of CD8A, CD8B, the TIL Z score, PD-L1, and the PD-L1/TIL Z score to predict clinical response to ICIs. 

TCGA dataset: We acquired available level-3 data published by TCGA, including 8634 samples with available survival information of 33 cancer types. Genomic somatic mutation data, copy number variation (CNV) data, mRNA expression data, and clinical information of each sample were downloaded from the GDC Data Portal (https://portal.gdc.cancer.gov, accessed on 30 April 2019). 

GEO dataset: A public mRNA high throughput sequencing dataset (GSE96058), containing sufficiently large numbers of breast cancer samples (*n* = 3069) deposited in GEO, was used to construct the validation cohort. The expressing matrix of mRNA plus clinical metadata were downloaded from GEO. Clinical metadata were used for Kaplan– Meier overall survival analysis, and mRNA expression profiles, which were constructed by GPL11154 of the Illumina HiSeq 2000 platform, were presented as fragments per kilobase of exon model per million mapped fragments (FPKM) and were transformed into TPM for transcriptome analysis.

### 4.2. Tumor-Infiltrating Lymphocyte Z Score

We calculated a comprehensive TIL score for each sample by applying an algorithmically optimized method, which was based on the expression of representative genes or gene sets of single samples from 26 determinants, consisting of 20 single factors (classified in MHC molecules, immunoinhibitors, and immunostimulators) and 6 immune cell types (activated CD4+ T cells, activated CD8+ T cells, effector memory CD4+ T cells, effector memory CD8+ T cells, Tregs, and MDSCs). The calculation was conducted through R code, developed by Charoentong et al. [42], and the source codes are available (https://github.com/mui-icbi/Immunophenogram, accessed on 20 May 2019). The RNA expression matrix was transformed into log_2_ (TPM+1) values and used as an input to calculate the comprehensive score of TILs. The result file generated by algorithm operation contained an average Z score and immunophenoscore (IPS); therefore, the Z score was selected as a TIL comprehensive score for further research. 

### 4.3. TIME Subtypes and Immune Cells Proportion

According to previous reports regarding the four TIME types [5], we stratified PD-L1 expression level and the TIL Z score into positive and negative groups: type I, PD-L1 positive with TIL positive; type II, PD-L1 negative with TIL negative; type III, PD-L1 positive with TIL negative; and type IV, PD-L1 negative with TIL positive, with a cut-off value of 90 percentile and median value, respectively. Additionally, a deconvolution approach [62], CIBERSORT, was applied to calculate the proportion of 22 immune cell types (https://cibersort.stanford.edu, accessed on 3 June 2019).

### 4.4. Genomic Analysis

The resulting data, consisting of detected somatic variants, was stored in mutation annotation format (MAF), and R package “Maftools” was used to summarize, analyze, annotate, and visualize MAF files in an efficient manner [63]. To evaluate TMB across samples, multiple somatic mutations, including nonsynonymous mutations, insertion-deletion mutations, and silent mutations, were counted and summated, with the exome size of 38 Mb, while germline mutations without somatic mutations were excluded [8]. The neoantigen number (*n* = 5,798) was evaluated by Vésteinn Thorsson et al. [64]. 

The data of amplification, deletion, and neutral status within a CNV threshold, recorded as “1”, “−1”, and “0”, respectively, was calculated by Gistic 2.0 [65]. mRNA expression profiles, which were constructed by the Illumina HiSeq V2 platform, were presented as counts and were transformed into transcripts per million (TPM) for analysis. Genes with multiple probes were represented by mean values of probes. 

### 4.5. Differential Gene Analysis and Pathway Score Analysis

In this part, each subtype was determined as an experimental group in turns, while others were set as control groups, and R package “edgeR” was subsequently performed on differential gene expression analysis [66,67]. Differential gene lists were identified with statistical significance (|log2FC| ≥ 2, FDR < 0.05). Pathways were downloaded from the Kyoto Encyclopedia of Genes and Genomes (KEGG), and Metascape (http://metascape.org, accessed on 14 July 2019) was performed on gene annotation and functional enrichment analysis with a significant threshold (*p* value < 0.01, enriched genes number ≥ 3) [68]. Gene and pathway scores were calculated via Python 3.7.1. Gene scores were computed by differential gene lists of each subtype in order to calculate which single pathway scores contributed to shared pathways. The calculation processes of each pathway score for type k (k = I, II, III, IV) was described as follows. 

For each gene, the mean log_2_FC score was firstly calculated across 4 subtypes:

(1) $${\mathrm{Mean}\;\log}_{2}\mathrm{FC}\;\mathrm{score}=\left(\sum_{k=I}^{\mathrm{IV}}\left|\log_{2}{\mathrm{FC}}_{k}\right|\right)/\;4,$$

where log2FCk is the log2 fold change score of type k (k = I, II, III, IV). If there is no such gene in the differential gene list of this subtype, then $\log_{2}{\mathrm{FC}}_{k}=0$.

Gene score of type k was then determined by an equation:

(2) $$\mathrm{Gene}\;\mathrm{score}=|\log_{2}{\mathrm{FC}}_{k}|\;/{\;\mathrm{Mean}\;\log}_{2}\mathrm{FC}\;\mathrm{score},$$

Mean log2FC score is the result of Equation (1).

Finally, the pathway score of type k was calculated:

(3) $$\mathrm{Pathway}\;\mathrm{score}=\sum \left(\mathrm{Gene}\;\mathrm{score}\right)\;/\;\sum_{k=I}^{\mathrm{IV}}{\left[\sum \left(\mathrm{Gene}\;\mathrm{score}\right)\right]}_{k},$$

where t∑ (Gene score) represents the total gene score at the same pathway of type k and $\sum_{k=I}^{\mathrm{IV}}{\left[\sum \left(\mathrm{Gene}\;\mathrm{score}\right)\right]}_{k}$ is the total gene score of that pathway from all subtypes. At the end, we visualized the result by using Power-BI (https://powerbi.microsoft.com) to plot a radar map of thr pathway score. 

### 4.6. Gene Set Variation Analysis (GSVA) Score of Gene Expression Signature

To compare the difference in the proportion of T cell exhaustion and MDSCs between four TIME subtypes, we used the gene set obtained for previous studies [69,70] to calculate the GSVA score across four subtypes. 

### 4.7. Survival Analysis

Univariate and multivariate logistic regression analyses were performed to determine significant factors of clinicopathologic characteristics. Patients who lacked follow-up or death time were pre-excluded when performing survival analyses. For categorical variables, such as TIME subtypes, PD-L1 expression status, and TIL status, a prognostic condition was estimated via Kaplan –Meier plots with a log-rank test and Cox proportional hazards regression analysis. Survival times were determined in months, from initial pathological diagnosis to death, or the last time the patient was known to be alive. *p* values less than 0.05 were considered statistically significant.

### 4.8. Statistical Analysis

R package “pROC” was used to plot the rate of response at various threshold settings of CD8A, CD8B, or the TIL Z score for generating the receiver operator characteristic (ROC) curve. Spearman rank correlation analysis was applied to compute the statistical significance of two continuous variables, which were exemplified as TMB, neoantigens, the TIL Z score, PD-L1 expression, and so on. One-way analysis of variance or a Wilcoxon rank sum test was applied for significance of differences between continuous values, which were listed as the immune cells proportion, tumor mutation burden, number of neoantigens, gene expression, such *IFNG* expression, and so on. Comparison of proportion according to categorical variables was performed using Pearson’s Chi-square test or the Fisher exact test. *p* values less than 0.05 were considered statistically significant.

## 5. Conclusions

In the current study, we developed a more robust method for classifying TIME subtypes at the big data analysis level and studied their characteristics shaping their corresponding microenvironments. It is noteworthy that the performance in the prognosis and prediction of the response to ICI immunotherapy of our method is superior to previous methods used in previous research. Considering the effectiveness, our classification method exhibits a better performance, which provides a potential option for clinical research and applications.

## Figures and Tables

**Table 1:** The sample size statistics and AUC value of different indicators for the immunotherapy research cohort.

Cohorts	Cancer Type	Drug	No. of Patients	No. of Responders	No. of Non-Responders	AUC Value				
						CD8A	CD8B	TIL (Z Score)	PD-L1	PD-L1/TIL
Hugo [28]	melanoma	anti-PD-1 (pembrolizumab and nivolumab)	26	13	13	0.503	0.497	0.686	0.598	0.722
Riaz [31]	melanoma	anti-PD-1 (nivolumab)	49	26	23	0.587	0.566	0.557	0.523	0.609
Miao [30]	ccRCC	anti-PD-1 (nivolumab)	33	20	13	0.554	0.488	0.515	0.415	0.658
Snyder [29]	urothelial cancer	anti-PD-L1 (atezolizumab)	25	9	16	0.646	0.632	0.611	0.59	0.611
Mariat- Hasan [32]	urothelial cancer	anti-PD-L1 (atezolizumab)	298	68	230	0.585	0.578	0.589	0.564	0.6

**Table 2:** Clinical, pathological, and molecular characteristics of pan-cancer, according to tumor immune microenvironment subtypes based on programmed death ligand 1 (PD-L1) expression and tumor infiltrating lymphocyte (TIL).

	Type I	Type II	Type III	Type IV	*p* Value
No.	280	3733	584	4037	
Age	56.22 ± 15.01	57.86 ± 14.87	61.84 ± 13.70	58.94 ± 13.72	9 × 10^−11^
Gender					0.0004998
Male	133 (47.50%)	1562 (41.84%)	303 (51.88%)	2077 (51.45%)	
Female	147 (52.50%)	2171 (58.16%)	281 (48.12%)	1960 (48.55%)	
Stage					0.0004998
I	40 (14.29%)	630 (16.88%)	157 (26.88%)	933 (23.11%)	
II	36 (12.86%)	749 (20.06%)	122 (20.89%)	815 (20.19%)	
III	41 (14.64%)	489 (13.10%)	107 (18.32%)	649 (16.08%)	
IV	34 (12.14%)	231 (6.19%)	61 (10.45%)	334 (8.27%)	
T cells	0.47 ± 0.18	0.28 ± 0.13	0.36 ± 0.14	0.36 ± 0.13	<2.2 × 10^−16^
B cells	0.08 ± 0.08	0.09 ± 0.09	0.10 ± 0.09	0.09 ± 0.10	0.0086
Macrophages	0.31 ± 0.17	0.46 ± 0.17	0.41 ± 0.14	0.37 ± 0.15	<2.2 × 10^−16^
DC cells	0.06 ± 0.06	0.04 ± 0.06	0.05 ±0.06	0.05 ± 0.06	<2.2 × 10^−16^
NK cells	0.04 ± 0.04	0.04 ± 0.04	0.04 ± 0.03	0.05 ± 0.04	<2.2 × 10^−16^
Mast cells	0.04 ± 0.04	0.08 ± 0.07	0.05 ± 0.04	0.07 ± 0.07	<2.2 × 10^−16^
Eosinophils	0.00 ± 0.00	0.00 ± 0.02	0.00 ± 0.01	0.00 ± 0.01	4.2 × 10^−11^
Neutrophils	0.00 ± 0.01	0.01 ± 0.02	0.01 ± 0.02	0.01 ± 0.02	2.1 × 10^−13^
TMB	4.22 ± 13.22	6.76 ± 30.72	6.85 ± 13.61	3.65 ± 12.33	1.8 × 10^−8^
Neoantigens	333.62 ± 1972.69	353.96 ± 1625.59	313.25 ± 677.87	187.79 ± 619.51	1.4 × 10^−5^
TP53-mut	65 (23.21%)	1409 (37.74%)	286 (48.97%)	1074 (26.60%)	<2.2 × 10^−16^
BRAF-mut	35 (12.50%)	151 (4.05%)	30 (5.14%)	297 (7.36%)	<2.2 × 10^−16^
HRAS-mut	13 (4.64%)	33 (0.88%)	20 (3.42%)	49 (1.21%)	8.734 × 10^−6^
IDH1-mut	6 (2.14%)	346 (9.27%)	11 (1.88%)	85 (2.11%)	<2.2 × 10^−16^
POLE-mut	4 (1.43%)	120 (3.21%)	27 (4.62%)	92 (2.28%)	<2.2 × 10^−16^
POLD1-mut	5 (1.79%)	65 (1.74%)	6 (1.03%)	38 (0.94%)	<2.2 × 10^−16^
PDCD1LG2 CNA					<2.2 × 10^−16^
Amplification	28 (10.00%)	114 (3.05%)	85 (14.55%)	88 (2.18%)	
Deletion	1 (0.36%)	166 (4.45%)	13 (2.23%)	101 (2.50%)	
PD-L1 CNA					<2.2 × 10^−16^
Amplification	28 (10.00%)	114 (3.05%)	84 (14.38%)	87 (2.16%)	
Deletion	1 (0.36%)	166 (4.45%)	13 (2.23%)	100 (2.48%)	
PDCD1 CNA					8.064 × 10^−5^
Amplification	0 (0.00%)	101 (2.71%)	9 (1.54%)	47 (1.16%)	
Deletion	34 (12.14%)	382 (10.23%)	89 (15.24%)	294 (7.28%)	
CTLA4 CNA					0.001178
Amplification	2 (0.71%)	136 (3.64%)	19 (3.25%)	88 (2.18%)	
Deletion	16 (5.71%)	149 (3.99%)	46 (7.88%)	125 (3.10%)	
Immuno-activating cytokines	2.81 ± 3.76	2.19 ± 3.49	4.52 ± 6.75	1.37 ± 2.37	<2.2 × 10^−16^
Immuno-suppressive cytokines	39.38 ± 33.96	39.24 ± 39.31	50.47±29.03	34.76 ± 37.71	<2.2 × 10^−16^
Cytolytic activity	34.68 ± 36.73	11.46 ± 19.96	47.71±72.69	12.15 ± 30.09	<2.2 × 10^−16^

Pan-cancer samples are divided into four groups based on PD-L1 expression and the TIL Z score as follows: type I, PD-L1 positive with TIL positive; type II, PD-L1 negative with TIL negative; type III, PD-L1 positive with TIL negative; and type IV, PD-L1 negative with TIL positive. The immuno-activating cytokines of each sample were calculated by using the mean values of interferon gamma, tumor necrosis factor, interleukin-12 subunit alpha, and interleukin-12 subunit beta. The immuno-suppressive cytokines of each sample were calculated by using the mean values of vascular endothelial growth factor A, transforming growth factor beta 1, interleukin 6, and interleukin 10. The cytolytic activity of each sample was calculated by using the mean values of granzyme and perforin 1. The predicted neoantigen number was referenced in a previous report written by Vésteinn Thorsson. Abbreviations: TMB, tumor mutation burden; TP53, tumor protein 53; BRAF, B-Raf Proto-Oncogene; HRAS, HRas proto-oncogene; IDH1, isocitrate dehydrogenase (NADP(+)) 1; POLE, DNA polymerase epsilon; POLD1, DNA polymerase delta 1; PDCD1LG2, programmed cell death 1 ligand 2; PD-L1, programmed death ligand 1; PDCD1, programmed cell death 1; CTLA4, cytotoxic T-lymphocyte-associated protein 4; CNA, copy number alteration; mut, mutation; x±σ, mean ± standard deviation.

**Table 3:** Univariate and multivariate cox proportional hazards analysis for overall survival in pan-cancer patients.

Variable	Univariate Prognostic Analysis			Multivariate Prognostic Analysis		
	HR	95% CI	*p*-Value	HR	95% CI	*p*-Value
Age > 60 years (vs. < 60 years)	1.87137	1.712–2.046	< 2 × 10^−16^	1.848602	1.5988–2.1374	<2 × 10^−16^
Gender, male (vs. female)	1.14972	1.054–1.255	0.002	1.159753	1.0093–1.3326	0.036577
Stage II (vs. stage I)	1.44218	1.219–1.706	1.89 × 10^−5^	1.326312	1.0929–1.6095	0.004236
Stage III (vs. stage I)	2.27638	1.934–2.679	<2 × 10^−16^	1.873979	1.5455–2.2723	1.69e-10
Stage IV (vs. stage I)	4.66921	3.957–5.509	<2 × 10^−16^	3.406277	2.7873–4.1627	<2 × 10^−16^
PD-L1 positive (vs. negative)	1.1452	0.9999–1.312	0.0501	————	—————	————
TIL positive (vs. negative)	0.69328	0.6345–0.7575	4 × 10^−16^	0.845795	0.7335–0.9752	0.021152
CD8+T high (vs. low)	0.7363	0.6744–0.8039	8.31 × 10^−12^	0.91313	0.7891–1.0567	0.222529
CD4+T activated high (vs. low)	1.1385	1.043–1.242	0.00355	1.071685	0.9069–1.2664	0.416427
Treg high (vs. low)	0.8552	0.7836–0.9333	0.000453	0.9463	0.8254–1.0849	0.428814
Macro M2 high (vs. low)	1.15472	1.058–1.26	0.00128	1.244084	1.0794–1.434	0.002581
Mast activated high (vs. low)	1.56816	1.422–1.73	<2 × 10^−16^	1.241577	1.0436–1.4771	0.014614
DC activated high (vs. low)	1.18640	1.086–1.296	0.000148	1.028179	0.8939–1.1827	0.697222
NK activated high (vs. low)	0.81109	0.7432–0.8852	2.66 × 10^−6^	1.272744	1.0986–1.4744	0.001312
B memory	1.20193	1.087–1.329	0.000331	1.200665	1.0121–1.4244	0.035958
TMB high (vs. low)	1.71388	1.559–1.884	<2 × 10^−16^	1.231722	0.9973–1.5213	0.053035
Neoantigens high (vs. low)	1.5202	1.361–1.698	1.01 × 10^−13^	1.029541	0.8496–1.2476	0.766486
TP53 mutation (vs. wild type)	1.72522	1.58–1.884	<2 × 10^−16^	1.321964	1.1383–1.5353	0.000255
BRAF mutation (vs. wild type)	0.4703	0.3531–0.6263	2.44 × 10^−7^	0.772879	0.5504–1.0854	0.136993
IDH1 mutation (vs. wild type)	0.6939	0.5493–0.8765	0.00218	1.211248	0.5724–2.5631	0.616285
POLE mutation (vs. wild type)	0.96719	0.7449–1.256	0.802	————	—————	————
POLD1 mutation (vs. wild type)	0.7212	0.4474–1.163	0.18	————	——————	————
PD-L1 amplification yes (vs. no)	1.4735	1.208–1.797	0.000128	1.026873	0.764–1.3802	0.860481
PDCD1 deletion yes (vs. no)	1.24219	1.084–1.424	0.00182	0.839484	0.6544–1.077	0.168638
CTLA4 deletion yes (vs. no)	1.44534	1.193–1.752	0.000173	1.007358	0.7006–1.4484	0.968436
Immuno-activating cytokines high (vs. low)	1.33658	1.224–1.46	1.1 × 10^−10^	0.987575	0.8485–1.1494	0.871716
Immuno-suppressive cytokines high (vs. low)	1.69775	1.552–1.857	<2 × 10^−16^	1.165356	1.0013–1.3563	0.048076
Cytolytic activity high (vs. low)	1.1153	1.022–1.217	0.0144	1.018329	0.8541–1.2141	0.839546

The immuno-activating cytokines of each sample were calculated by using the mean value of interferon gamma, tumor necrosis factor, interleukin-12 subunit alpha, and interleukin-12 subunit beta. The immuno-suppressive cytokines of each sample were calculated by using the mean value of vascular endothelial growth factor A, transforming growth factor beta 1, interleukin 6, and interleukin 10. The cytolytic activity of each sample was calculated by using the mean value of granzyme and perforin 1. The predicted neoantigen number was referenced in a previous report written by Vésteinn Thorsson. “High” means the value is higher than the median, and “low” means the opposite. Abbreviations: HR: hazard ratio; 95% CI: 95% confidence interval; PD-L1, programmed death ligand 1; TIL: tumor infiltrating lymphocyte; Macro, macrophages; TMB, tumor mutation burden; TP53, tumor protein 53; BRAF, B-Raf Proto-Oncogene; IDH1, isocitrate dehydrogenase 1; POLE, DNA polymerase epsilon; POLD1, DNA polymerase delta 1; PDCD1, programmed cell death 1; CTLA4, cytotoxic T-lymphocyte-associated protein 4.

## Data Availability

Data is contained within the article or supplementary material.

## Supplementary Materials

The following are available online at https://www.mdpi.com/article/10.3390/ijms22105158/s1. Figure S1: Based on survival analysis of positive vs. negative PD-L1 or TIL subgroups to classify samples. (A) The value distribution of PD-L1 expression across 33 cancer types. (B) Survival analysis of positive vs. negative PD-L1 subgroups in each cut-point. (C) The value distribution of TIL status across 33 cancer types. (D) Survival analysis of positive vs. negative TIL subgroups in each cut-point. (E) Correlation relationship between TIL status and PD-L1 expression. (F) Response rate to ICI immunotherapy of four TIME subtypes. (G) The proportions of 4 TIME subtypes across 33 cancer types. Figure S2: Genomic characterization between four subtypes. (A) The correlation between tumor mutation burden and PD-L1 expression. (B) The correlation between neoantigens and PD-L1 expression. (C) Difference in TIL between TP53 mutation and wild type. (D) The samples proportion of TIL+ and TIL− between TP53 mutation and wild type. (E) Somatic mutational interactions among 4 subtypes. (F) The oncogene pattern in each subtype. (G) Difference in TIL between BRAF mutation and wild type. (H) The samples proportion of TIL+ and TIL− between BRAF mutation and wild type. (I) Difference in TIL between HRAS mutation and wild type. (J) Difference in PD-L1 expression between IDH1 mutation and wild type. ****, *p* < 0.0001; ***, *p* < 0.001; **, *p* < 0.01; *, *p* < 0.05. Figure S3: The transcriptomic patterns discrepancy in four TIME subtypes. (A) Difference in PD-L1 expression between PDCD1LG2 amplification and not amplification. (B) Difference in PD-L1 expression between PD-L1 amplification and not amplification. (C) Difference in PD-L1 expression between PDCD1 deletion and not deletion. (D) Difference in PD-L1 expression between CTLA4 deletion and not deletion. (E) The gene expression distributions of cytokines and cytolysis factors in each subtype. (F) The gene expression distributions of growth factors and receptors in each subtype. (G) The gene expression distributions of growth factors and receptors between TIL positive and TIL negative samples. (H) The correlation coefficient between the TIL score and expression of growth factors, as well as receptors. ****, *p* < 0.0001; ***, *p* < 0.001; **, *p* < 0.01; *, *p* < 0.05. Figure S4: Survival analysis of positive vs. negative PD-L1 or TIL subgroups in the validation cohort from the Gene Expression Omnibus database. (A) Survival analysis of positive vs. negative PD-L1 subgroups in each cut-point. (B) Survival analysis of positive vs. negative TIL subgroups in each cut-point. Figure S5: The composition and abundance of immune cell types and expression distribution among four TIME subtypes in validation cohort from the Gene Expression Omnibus database. (A) Survival analysis of type I, type II, type III, and type IV. (B) The proportions of patients in type I, type II, type III, and type IV. (C) The abundance difference among 8 types of immune cells within 4 subtypes. (D) The abundance difference of 6 main subclass in each subtype. (E) The gene expression distributions of growth factors and receptors in each subtype. (F) The gene expression distributions of growth factors and receptors between TIL positive and TIL negative samples. (G) The correlation coefficient between the TIL score and expression of growth factors, as well as receptors. ****, *p* < 0.0001; ***, *p* < 0.001; **, *p* < 0.01; *, *p* < 0.05. Table S1: The response rate to ICI immunotherapy among four subtypes. Table S2: Statistical data of PD-L1 TPM, TIL (Z score), and subtypes proportion across pan-cancer. Table S3: Statistical data of 8 immune cell proportion across the four subtypes of tumor immune microenvironment. Table S4: The relative proportion of 20 immune cell subtypes across the four subtypes of tumor immune microenvironment. Table S5: Statistical data of the log2 value of TMB and the log2 value of neoantigens across the four subtypes of tumor immune microenvironment. Table S6: Mutation rate of the top 10 genes across the four subtypes of tumor immune microenvironment. Table S7: The drive gene detected of the four subtypes of tumor immune microenvironment. Table S8: Statistical data of 75 immune related genes across the four subtypes of tumor immune microenvironment. Table S9: The pathway scores of the shared pathways across the four subtypes of tumor immune microenvironment. Table S10: Statistical data of the specific gene TPM across the four subtypes of tumor immune microenvironment. Table S11: Statistical data of 8 immune cell proportion across the four subtypes of tumor immune microenvironment in the validation cohort from the Gene Expression Omnibus database. Table S12: The relative proportion of 20 immune cell subtypes across the four subtypes of tumor immune microenvironment in the validation cohort from the Gene Expression Omnibus database. Table S13: Statistical data of the specific gene TPM across the four subtypes of tumor immune microenvironment in the validation cohort from the Gene Expression Omnibus database.

## Abbreviations

ICI	Immune checkpoint inhibitors;
TIME	Tumor immune microenvironment;
PD-1	Programmed death1;
PD-L1	Programmed death ligand 1;
TCGA	The Cancer Genome Atlas;
TIL	Tumor-infiltrating lymphocyte;
TMB	Tumor mutation burden;
IHC	Immunohistochemistry;
THYM	Thymoma;
UCS	Uterine carcinosarcoma;
LUSC	Lung squamous cell carcinoma;
LIHC	Liver hepatocellular carcinoma;
TP53	Tumor protein 53;
TTN	Titin;
LRP1B	LDL receptor related protein 1B;
CSMD3	CUB and Sushi Multiple Domains 3;
BRAF	B-Raf Proto-Oncogene;
FAT1	FAT Atypical Cadherin 1;
GTF2I	General Transcription Factor Iii;
PCLO	Piccolo;
ZFHX4	Zinc Finger Homeobox 4;
SPTA1	Spectrin alpha, erythrocytic 1;
APC	Adenomatous polyposis coli;
KMT2D	Lysine methyltransferase 2D;
PIK3CA	Phosphatidylinositol-4,5-bisphosphate 3-kinase catalytic subunit alpha;
PDCD1LG2	Programmed Cell Death 1 Ligand 2;
VTCN1	V-set domain containing T cell activation inhibitor 1;
PDCD1	Programmed Cell Death 1;
CTLA4	Cytotoxic T-lymphocyte-associated protein 4;
DEG	Different expressed gene;
KEGG	Kyoto Encyclopedia of Genes and Genomes;
IFNG	Interferon gamma;
TNF	Tumor necrosis factor;
TNFA	Tumor Necrosis Factor Alpha;
IL6	Interleukin 6;
IL12	Interleukin 12;
IL12A	Interleukin 12A;
IL12B	Interleukin 12B;
IL10	Interleukin 10;
GZMB	Granzyme B;
PRF1	Perforin-1;
KRAS	Kirsten ras;
VEGFA	Vascular endothelial growth factor A;
TGFB1	Transforming growth factor beta 1;
HRAS	HRas proto-oncogene;
IDH1	Isocitrate dehydrogenase (NADP(+)) 1;
POLE	DNA polymerase epsilon;
POLD1	DNA polymerase delta 1;
MUC16	Mucin 16;
RYR2	Ryanodine receptor 2;
SYNE1	Spectrin repeat containing nuclear envelope protein 1;
FLG	Filaggrin;
USH2A	Usherin;
CDKN2A	Cyclin dependent kinase inhibitor 2A;
MB21D2	Mab-21 domain containing 2;
NDUFA13	NADH:ubiquinone oxidoreductase subunit A13;
DGCR6L	DiGeorge syndrome critical region gene 6 like;
S100A1	S100 calcium binding protein A1;
IAPP	Islet amyloid polypeptide;
SLC3A2	Solute carrier family 3 member 2;
KLF3	Kruppel like factor 3;
GNG12	G protein subunit gamma 12;
NRAS	NRAS proto-oncogene;
RAB9B	RAB9B, member RAS oncogene family;
SH3BGRL2	SH3 domain binding glutamate rich protein like 2;
TNP1	Transition protein 1;
RPL22	Ribosomal protein L22;
MRPL22	Mitochondrial ribosomal protein L22;
CBLN3	Cerebellin 3 precursor;
PAIP2	Poly(A) binding protein interacting protein 2;
SEC61B	SEC61 translocon subunit beta;
DBI	Diazepam binding inhibitor;
GNA11	G protein subunit alpha 11;
ARHGAP1	Rho GTPase activating protein 1.
