# Supplementary material for: Multi-Omics Perspective Reveals the Different Patterns of Tumor Immune Microenvironment Based on Programmed Death Ligand 1 (PD-L1) Expression and Predictor of Responses to Immune Checkpoint Blockade across Pan-Cancer
Source: Int J Mol Sci. 2021 May 13;22(10):5158. doi: 10.3390/ijms22105158 (PMC8153013; doi:10.3390/ijms22105158)
Supplement: Supplementary file 1 [file ijms-22-05158-s001.zip › Supplementary Table.pdf]

Table S1. The response rate to ICI immunotherapy among four subtypes.

Cohorts	Cancer type	Drug	No. of patients	Response rate	Type I (R, NR, %)	Type II (R, NR, %)	Type III (R, NR, %)	Type IV (R, NR, %)
Hugo	melanoma	anti-PD-1 (pembrolizumab and nivolumab)	26	50%	1, 0, 100%	4, 7, 36.36%	1, 1, 50%	7, 5, 58.33%
Riaz	melanoma	anti-PD-1 (nivolumab)	49	53.06%	3, 0, 100%	11, 11, 50%	1, 1, 50%	11, 11, 50%
Miao	ccRCC	anti-PD-1 (nivolumab)	33	60.6%	0, 1, 0%	9, 4, 69.23%	0, 3, 0%	11, 5, 68.75%
Snyder	urothelial cancer	anti-PD-L1 (atezolizumab)	25	36%	0, 0	2, 7, 22.22%	1, 2, 33.33%	6, 7, 46.15%
Mariathasan	urothelial cancer	anti-PD-L1 (atezolizumab)	298	22.82%	3, 5, 37.5%	21, 106, 16.54%	4, 18, 18.18%	40, 101, 28.37%

R: Responders; NR: Non-responders; %: Response rate.

Table S2. Statistical data of PD-L1 TPM, TIL (Z score) and subtypes proportion across pan-cancer.

Cancer	PD-L1 TPM		TIL (Z score)		Subtypes proportion (%)				
	x±σ	p value	x±σ	p value	Type I	Type II	Type III	Type IV	p value
**UCS**	1.1965±1.5641		2.5129±0.3234		0	88.24	0	11.76	<2.2e-16
**ACC**	1.5098±4.0215		3.2289±0.5038		0	31.58	1.32	67.11	<2.2e-16
**LIHC**	1.2585±1.7312		3.3436±0.3922		0	15.87	0	84.13	<2.2e-16
**LAML**	1.4913±2.1768		2.7441±0.3582		0	72.31	0.77	26.92	<2.2e-16
**UVM**	1.7863±1.901		3.2651±0.3378		0	18.57	0	81.43	<2.2e-16
**UCEC**	2.0186±2.9133		2.9671±0.3904		0.41	46.75	0.41	52.44	<2.2e-16
**PRAD**	1.5402±1.3266		3.117±0.2414		0.2	25.56	0	74.24	<2.2e-16
**LGG**	2.5774±4.0419		2.7171±0.2654		0.63	80.76	1.9	16.7	<2.2e-16
**OV**	2.2147±2.3987		2.7622±0.3614		0.29	71.97	0	27.75	<2.2e-16
**READ**	2.7316±3.6447		3.1745±0.3236		0.89	23.21	0.89	75	<2.2e-16
**CHOL**	4.1926±9.495		3.1718±0.411		0	26.47	2.94	70.59	<2.2e-16
**COAD**	3.6617±6.3532		3.1834±0.3787		0.95	23.17	1.9	73.97	<2.2e-16
**SARC**	5.5631±11.241		2.7278±0.2961		0.87	71.43	8.23	19.48	<2.2e-16
**SKCM**	4.975±10.2462		3.2449±0.4173		5.33	26.67	1.33	66.67	<2.2e-16
**BLCA**	9.3797±16.0506		3.038±0.3935		5.52	33.13	12.88	48.47	6.466e-10
**BRCA**	4.3186±5.3206		2.8286±0.2941		1.11	64.78	2.53	31.58	<2.2e-16
**PAAD**	3.6685±2.6827		2.9688±0.3212		0	48.59	1.41	50	<2.2e-16
**KIRP**	5.4884±7.6951	<2.2e-16	3.1708±0.3268	<2.2e-16	3.08	18.85	5	73.08	<2.2e-16
**STAD**	8.3054±35.2599		3.0643±0.388		3.27	32.73	4.36	59.64	<2.2e-16
**MESO**	10.293±19.6711		2.8601±0.321		2.9	44.93	15.94	36.23	1.703e-09
**GBM**	5.9626±6.9316		2.6967±0.295		0.76	72.52	7.63	19.08	<2.2e-16
**ESCA**	6.3593±8.6932		2.9069±0.3929		1.61	50	6.45	41.94	1.496e-15
**TGCT**	6.3926±7.1982		2.5819±0.2161		0	84.85	9.09	6.06	<2.2e-16
**KIRC**	6.0137±12.0815		3.1185±0.3061		1.38	27.17	2.17	69.29	<2.2e-16
**PCPG**	6.2965±5.602		3.0592±0.2854		3.37	32.02	3.37	61.24	<2.2e-16
**KICH**	12.264±30.428		3.3485±0.4434		7.94	11.11	3.17	77.78	<2.2e-16
**LUAD**	13.2727±19.7916		2.7577±0.3256		1.41	52.69	20.84	25.06	1.351e-11
**THCA**	7.8647±5.5019		3.1534±0.2747		7.26	18.35	3.43	70.97	<2.2e-16
**CESC**	15.4899±24.5242		3.0574±0.4021		13.5	21.9	16.42	48.18	1.307e-06
**HNSC**	14.1869±23.0509		2.9816±0.3232		9.68	30.41	16.67	43.24	6.997e-06
**LUSC**	19.5927±27.5279		2.5727±0.277		1.4	57.94	31.78	8.88	<2.2e-16
**DLBC**	24.2074±33.5574		3.0615±0.4692		7.14	4.76	30.95	57.14	1.682e-15
**THYM**	32.8036±28.7492		3.1237±0.2366		55.26	7.02	13.16	24.56	6.306e-12

Pan-cancer samples are divided into four groups based on PD-L1 expression and TIL Z score as follows:

type Ⅰ, PD-L1 positive with TIL positive; type Ⅱ, PD-L1 negative with TIL negative; type Ⅲ, PD-L1

positive with TIL negative; and type Ⅳ, PD-L1 negative with TIL positive. Abbreviations: TPM, transcripts per million reads; x±σ, mean ± standard deviation; PD-L1, programmed death ligand 1; TIL: tumor infiltrating lymphocyte; UCS, uterine carcinosarcoma; ACC, adrenocortical carcinoma; LIHC, liver hepatocellular carcinoma; LAML, acute myeloid leukemia; UVM, uveal melanoma; UCEC, uterine corpus endometrial carcinoma; PRAD, prostate adenocarcinoma; LGG, brain lower grade glioma; OV, ovarian serous cystadenocarcinoma; READ, rectum adenocarcinoma; CHOL, cholangiocarcinoma; COAD, colon adenocarcinoma; SARC, sarcoma; SKCM, skin cutaneous melanoma; BLCA, bladder urothelial carcinoma; BRCA, breast invasive carcinoma; PAAD, pancreatic adenocarcinoma; KIRP, kidney renal papillary cell carcinoma; STAD, stomach adenocarcinoma; MESO, mesothelioma; GBM, glioblastoma multiforme; ESCA, esophageal carcinoma; TGCT, testicular germ cell tumors; KIRC, kidney renal clear cell carcinoma; PCPG, pheochromocytoma and paraganglioma; KICH, kidney chromophobe; LUAD, lung adenocarcinoma; THCA, thyroid carcinoma; CESC, cervical squamous cell carcinoma and endocervical adenocarcinoma; HNSC, head and neck squamous cell carcinoma; LUSC, lung squamous cell carcinoma; DLBC, lymphoid neoplasm diffuse large b-cell lymphoma; THYM, thymoma.

Table S3. Statistical data of 8 immune cell proportion across the four subtypes of tumor immune microenvironment.

Immune cell types	x±σ				
	**Type I**	**Type II**	**Type III**	**Type IV**	**p value**
**T cells**	0.466±0.1833	0.2817±0.1255	0.3569±0.135	0.3582±0.134	<2.2e-16
**B cells**	0.0773±0.0831	0.0894±0.0944	0.0953±0.0912	0.0939±0.0963	0.0086
**Macrophages**	0.3067±0.1662	0.4638±0.1656	0.4054±0.1406	0.375±0.1508	<2.2e-16
**DC cells**	0.0622±0.0571	0.0372±0.0572	0.0497±0.0586	0.0452±0.0608	<2.2e-16
**NK cells**	0.0418±0.0374	0.0389±0.0357	0.0358±0.0297	0.0471±0.0384	<2.2e-16
**Mast cells**	0.0421±0.0409	0.0772±0.0736	0.0486±0.0413	0.0741±0.0661	<2.2e-16
**Eosinophils**	0.0004±0.0026	0.0033±0.0196	0.001±0.005	0.0013±0.0079	4.2e-11
**Neutrophils**	0.0036±0.0084	0.0085±0.0222	0.0074±0.0179	0.0053±0.017	2.1e-13

Pan-cancer samples are divided into four groups based on PD-L1 expression and TIL Z score as follows: type I, PD-L1 positive with TIL positive; type II, PD-L1 negative with TIL negative; type III, PD-L1 positive with TIL negative; and type IV, PD-L1 negative with TIL positive. Abbreviations: x±σ, mean ± standard deviation; DC cells, dendritic cells; NK cells, Natural killer cells.

Table S4. The relative proportion of 20 immune cell subtypes across the four subtypes of tumor immune microenvironment.

Immune cell subtypes	Relative proportion (%)			
	Type I	Type II	Type III	Type IV
**T cells CD8**	43.56	27.44	39.43	33.81
**T cells CD4 naive**	3.36	1.68	0.4	1
**T cells CD4 memory resting**	15.03	41.49	24.86	36.15
**T cells CD4 memory activated**	10.3	6.03	16.04	5.16
**T cells follicular helper**	14.16	11.15	9.88	10.05
**T cells gamma delta**	1.48	1.69	1.35	2.02
**T cells regulatory (Tregs)**	12.11	10.53	8.05	11.82
**B cells naive**	50.81	61.73	55.15	56.22
**B cells memory**	7.46	5.64	4.5	8.4
**Plasma cells**	41.73	32.63	40.35	35.38
**Dendritic cells resting**	71.93	60.09	66.08	61.98
**Dendritic cells activated**	28.07	39.91	33.92	38.02
**NK cells resting**	36.69	36.63	50.24	25.44
**NK cells activated**	63.31	63.37	49.76	74.56
**Mast cells resting**	79.7	74.71	75.67	85.55
**Mast cells activated**	20.3	25.29	24.33	14.45
**Monocytes**	4.64	12.59	5.21	7.05
**Macrophages M0**	32.35	35.68	36.48	33.81
**Macrophages M1**	29.13	11.86	22.44	17.28
**Macrophages M2**	33.88	39.88	35.87	41.86

Pan-cancer samples are divided into four groups based on PD-L1 expression and TIL Z score as follows: type I, PD-L1 positive with TIL positive; type II, PD-L1 negative with TIL negative; type III, PD-L1 positive with TIL negative; and type IV, PD-L1 negative with TIL positive. The relative proportion refers to the proportion of each immune cell subtype to the immune cell type which it belongs.

Table S5. Statistical data of the log2 value of TMB and the log2 value of Neoantigens across the four subtypes of tumor immune microenvironment.

Genomic factors	x±σ				
	**Type I**	**Type II**	**Type III**	**Type IV**	**p value**
**log_2(TMB)**	0.3404±2.1294	0.6836±1.9553	1.6336±1.9317	0.4576±1.8091	<2.2e-16
**log_2(Neoantigens)**	6.2054±2.1765	6.3903±1.9487	7.3379±1.7319	6.2546±1.7246	<2.2e-16

Pan-cancer samples are divided into four groups based on PD-L1 expression and TIL Z score as follows: type I, PD-L1 positive with TIL positive; type II, PD-L1 negative with TIL negative; type III, PD-L1 positive with TIL negative; and type IV, PD-L1 negative with TIL positive. Abbreviations: log2(TMB), the log2 value of TMB; log2(Neoantigens), the log2 value of neoantigens; x±σ, mean ± standard deviation.

Table S6. Mutation rate of the top ten genes across the four subtypes of tumor immune microenvironment.

Genes	Mutation rate			
	Type I	Type II	Type III	Type IV
TTN	29%	29%	46%	26%
TP53	24%	41%	51%	29%
MUC16	16%	18%	28%	16%
PIK3CA	14%	14%	——	12%
BRAF	13%	——	——	——
CSMD3	11%	13%	25%	9%
LRP1B	10%	11%	23%	9%
FAT1	10%	——	——	——
GTF2I	10%	——	——	——
PCLO	9%	——	——	——
RYR2	13%	13%	22%	9%
SYNE1	——	12%	——	9%
FLG	——	11%	17%	——
USH2A	——	10%	23%	——
ZFHX4	——	——	20%	——
SPTA1	——	——	17%	——
APC	——	——	——	9%
KMT2D	——	——	——	8%

Pan-cancer samples are divided into four groups based on PD-L1 expression and TIL Z score as follows: type I, PD-L1 positive with TIL positive; type II, PD-L1 negative with TIL negative; type III, PD-L1 positive with TIL negative; and type IV, PD-L1 negative with TIL positive. Abbreviations: TTN, titin; TP53, tumor protein 53; MUC16, mucin 16; PIK3CA, phosphatidylinositol-4,5-bisphosphate 3-kinase catalytic subunit alpha; BRAF, B-Raf Proto-Oncogene; CSMD3, CUB and Sushi Multiple Domains 3; LRP1B, LDL receptor related protein 1B; FAT1, FAT Atypical Cadherin 1; GTF2I, general transcription

factor Iii; PCLO, piccolo presynaptic cytomatrix protein; RYR2, ryanodine receptor 2; SYNE1, spectrin repeat containing nuclear envelope protein 1; FLG, filaggrin; USH2A, usherin; ZFHX4, zinc finger homeobox 4; SPTA1, spectrin alpha, erythrocytic 1; APC, adenomatous polyposis coli; KMT2D, lysine methyltransferase 2D.

Table S7. The drive gene detected of the four subtypes of tumor immune microenvironment.

Drive genes			
**Type I**	**Type II**	**Type III**	**Type IV**
GTF2I	——	——	——
BRAF	——	——	BRAF
KRAS	KRAS	KRAS	KRAS
HRAS	——	——	HRAS
CDKN2A	——	——	——
MB21D2	——	——	——
PIK3CA	——	——	——
——	IDH1	——	IDH1
——	NDUFA13	——	——
——	DGCR6L	——	——
——	S100A1	——	——
——	IAPP	——	——
——	——	SLC3A2	——
——	——	KLF3	——
——	——	GNG12	——
——	——	NRAS	NRAS
——	——	RAB9B	——
——	——	——	SH3BGRL2
——	——	——	TNP1
——	——	——	RPL22
——	——	——	MRPL22
——	——	——	CBLN3
——	——	——	PAIP2
——	——	——	SEC61B
——	——	——	DBI
——	——	——	GNA11
——	——	——	ARHGAP1
——	——	——	TVP23A

Pan-cancer samples are divided into four groups based on PD-L1 expression and TIL Z score as follows: type I, PD-L1 positive with TIL positive; type II, PD-L1 negative with TIL negative; type III, PD-L1 positive with TIL negative; and type IV, PD-L1 negative with TIL positive. Drive genes is detected by a function oncodrive from R package "maftools", which is based on algorithm oncodriveCLUST which was originally implemented in Python. Abbreviations: GTF2I, general transcription factor Iii; BRAF, B-

Raf Proto-Oncogene; KRAS, kirsten ras; HRAS, HRas proto-oncogene; CDKN2A, cyclin dependent kinase inhibitor 2A; MB21D2, Mab-21 domain containing 2; PIK3CA, phosphatidylinositol-4,5-bisphosphate 3-kinase catalytic subunit alpha; IDH1, isocitrate dehydrogenase (NADP(+)) 1; NDUFA13, NADH:ubiquinone oxidoreductase subunit A13; DGCR6L, DiGeorge syndrome critical region gene 6 like; S100A1, S100 calcium binding protein A1; IAPP, islet amyloid polypeptide; SLC3A2, solute carrier family 3 member 2; KLF3, Kruppel like factor 3; GNG12, G protein subunit gamma 12; NRAS, NRAS proto-oncogene; RAB9B, RAB9B, member RAS oncogene family; SH3BGRL2, SH3 domain binding glutamate rich protein like 2; TNP1, transition protein 1; RPL22, ribosomal protein L22; MRPL22, mitochondrial ribosomal protein L22; CBLN3, cerebellin 3 precursor; PAIP2, poly(A) binding protein interacting protein 2; SEC61B, SEC61 translocon subunit beta; DBI, diazepam binding inhibitor; GNA11, G protein subunit alpha 11; ARHGAP1, Rho GTPase activating protein 1; TVP23A, trans-golgi network vesicle protein 23 homolog A.

Table S8. Statistical data of 75 immune related genes across the four subtypes of tumor immune microenvironment.

Genes	log10(TPM median + 1)				Amplification frequency				Deletion frequency			
	Type I	Type II	Type III	Type IV	Type I	Type II	Type III	Type IV	Type I	Type II	Type III	Type IV
**CD80**	0.3203	0.2187	0.5281	0.1387	-0.0138	0.0101	0.0097	-0.0098	-0.0019	0.0043	0.0160	-0.0062
**CD28**	0.4961	0.3693	0.5735	0.2757	-0.0215	0.0081	0.0039	-0.0066	0.0184	0.0005	0.0401	-0.0076
**ICOSLG**	0.1018	0.1332	0.1294	0.1010	-0.0161	0.0093	-0.0081	-0.0063	-0.0081	0.0036	0.0033	-0.0033
**PDCD1LG2**	0.9779	0.6180	1.2218	0.4172	0.0632	-0.0060	0.1088	-0.0147	-0.0290	0.0119	-0.0103	-0.0075
**CD274**	1.3548	0.5686	1.4731	0.5148	0.0634	-0.0057	0.1073	-0.0147	-0.0289	0.0120	-0.0102	-0.0077
**VTCN1**	0.1741	0.5848	0.4184	0.2942	-0.0155	0.0078	-0.0057	-0.0054	-0.0068	0.0064	0.0290	-0.0097
**SLAMF7**	1.0834	0.7382	1.3993	0.6645	-0.0145	0.0163	0.0104	-0.0156	-0.0073	0.0015	-0.0005	-0.0009
**BTN3A2**	1.5395	1.2574	1.4813	1.3310	0.0069	0.0064	-0.0001	-0.0064	-0.0172	0.0063	0.0117	-0.0064
**BTN3A1**	1.4734	1.2863	1.4860	1.2852	0.0071	0.0066	0.0002	-0.0067	-0.0172	0.0063	0.0117	-0.0064
**C10orf54**	1.5927	1.4480	1.5955	1.3713	-0.0239	0.0087	0.0050	-0.0071	-0.0070	0.0002	0.0045	-0.0004
**CD276**	1.6237	1.8152	1.8457	1.6625	-0.0032	0.0037	-0.0025	-0.0029	0.0008	0.0036	-0.0102	-0.0019
**TNFSF9**	0.8055	0.6094	1.0038	0.5845	0.0011	0.0072	0.0004	-0.0068	-0.0241	0.0105	-0.0135	-0.0061
**TNF**	0.5080	0.4993	0.6486	0.3278	0.0102	0.0005	-0.0005	-0.0011	-0.0183	0.0077	0.0176	-0.0084
**TNFSF4**	0.5958	0.6049	0.7296	0.4572	-0.0182	0.0127	-0.0079	-0.0094	0.0013	0.0021	-0.0008	-0.0020
**IL1B**	0.8064	0.7561	1.0571	0.5805	-0.0198	0.0071	0.0074	-0.0063	-0.0052	0.0029	-0.0073	-0.0013
**CXCL9**	1.9398	1.1779	2.0973	1.1652	-0.0148	0.0154	0.0081	-0.0145	-0.0031	0.0028	-0.0035	-0.0019
**CXCL10**	2.1239	1.4458	2.2517	1.4066	-0.0147	0.0153	0.0083	-0.0144	-0.0031	0.0028	-0.0035	-0.0019
**CCL5**	2.0905	1.5293	2.1492	1.5699	-	0.0025	-0.0035	-0.0012	-	0.0118	-	-

					0.0097				0.0247		0.0079	0.0081
**VEGFB**	2.1423	2.2064	2.1531	2.1869	0.0025	0.0134	0.0021	-0.0130	-0.0109	0.0055	-0.0061	-0.0035
**CX3CL1**	1.5736	1.4184	1.6616	1.4120	-0.0065	0.0019	0.0085	-0.0025	-0.0015	0.0072	-0.0091	-0.0052
**TGFB1**	1.9251	1.8305	2.0088	1.6852	-0.0179	0.0051	0.0346	-0.0085	-0.0262	0.0106	0.0127	-0.0098
**VEGFA**	1.4662	1.4905	1.5972	1.4913	-0.0109	0.0059	0.0030	-0.0052	-0.0081	0.0052	-0.0031	-0.0038
**CD70**	0.5859	0.2989	0.6044	0.3016	0.0010	0.0070	0.0020	-0.0069	-0.0237	0.0111	-0.0131	-0.0068
**CD40LG**	0.4312	0.2399	0.3926	0.2615	0.0105	0.0041	0.0138	-0.0066	-0.0115	0.0066	-0.0031	-0.0049
**IL10**	0.3088	0.2937	0.4467	0.1616	-0.0208	0.0105	-0.0047	-0.0076	-0.0044	0.0055	-0.0050	-0.0040
**IFNG**	0.4397	0.0956	0.5770	0.1034	-0.0272	0.0126	0.0098	-0.0113	-0.0053	0.0039	-0.0004	-0.0032
**IL1A**	0.1944	0.1882	0.6286	0.0934	-0.0194	0.0068	0.0078	-0.0061	-0.0054	0.0026	-0.0076	-0.0010
**IL12A**	0.1982	0.2505	0.3109	0.1409	-0.0137	0.0084	0.0452	-0.0135	-0.0041	0.0040	0.0007	-0.0036
**IFNA2**	0.0000	0.0000	0.0000	0.0000	0.0047	0.0029	0.0175	-0.0055	-0.0074	0.0184	0.0227	-0.0198
**IFNA1**	0.0000	0.0000	0.0000	0.0000	0.0048	0.0027	0.0193	-0.0057	-0.0094	0.0184	0.0241	-0.0199
**IL4**	0.0586	0.0509	0.0404	0.0450	-0.0059	0.0008	-0.0274	0.0036	-0.0119	0.0036	0.0028	-0.0029
**IL2**	0.0309	0.0000	0.0382	0.0167	-0.0012	0.0054	-0.0002	-0.0049	-0.0098	0.0050	-0.0021	-0.0037
**IL13**	0.0217	0.0160	0.0238	0.0098	-0.0061	0.0011	-0.0276	0.0034	-0.0117	0.0037	0.0029	-0.0030
**TNFRSF18**	1.1031	0.8110	1.2711	0.7118	-0.0263	0.0145	0.0041	-0.0122	-0.0484	0.0115	0.0048	-0.0081
**TIGIT**	0.6805	0.3483	0.8267	0.3037	-0.0187	0.0102	0.0116	-0.0099	-0.0009	0.0032	0.0102	-0.0044
**PDCD1**	0.9511	0.4209	0.9007	0.4105	-0.0182	0.0089	-0.0028	-0.0065	0.0285	0.0098	0.0596	-0.0197
**CTLA4**	0.7928	0.4011	0.9165	0.3619	-0.0213	0.0080	0.0041	-0.0066	0.0180	0.0010	0.0397	-0.0080
**IL2RA**	0.7189	0.5409	1.0712	0.3913	-0.0083	0.0131	-0.0080	-0.0105	0.0032	0.0025	0.0008	-0.0027
**TNFRSF4**	0.9307	0.7939	0.9990	0.7702	-0.0263	0.0145	0.0041	-0.0122	-0.0484	0.0115	0.0048	-0.0081
**CD27**	1.2473	0.7632	1.2527	0.7593	-	0.0163	0.0054	-0.0144	-	0.0038	0.0067	-

					0.0210				0.0185			0.0032
**LAG3**	1.0336	0.6141	1.1296	0.5641	-0.0167	0.0159	0.0061	-0.0145	-0.0241	0.0029	0.0082	-0.0022
**TNFRSF9**	0.4974	0.2787	0.6772	0.2172	-0.0276	0.0125	0.0047	-0.0104	-0.0492	0.0112	0.0038	-0.0075
**ICOS**	0.7432	0.2714	0.7143	0.2374	-0.0209	0.0076	0.0044	-0.0062	0.0178	0.0016	0.0395	-0.0084
**BTLA**	0.2973	0.1416	0.3076	0.1121	-0.0170	0.0103	0.0099	-0.0099	-0.0033	0.0006	0.0131	-0.0023
**KIR2DL3**	0.0348	0.0119	0.0358	0.0138	-0.0120	0.0075	0.0245	-0.0097	-0.0218	0.0137	0.0083	-0.0124
**KIR2DL1**	0.0323	0.0152	0.0376	0.0161	-0.0120	0.0075	0.0245	-0.0097	-0.0218	0.0137	0.0083	-0.0124
**TNFRSF14**	1.5573	1.3036	1.4711	1.4984	-0.0263	0.0145	0.0041	-0.0122	-0.0484	0.0115	0.0048	-0.0081
**EDNRB**	0.7013	0.8715	0.7557	0.9276	0.0052	0.0089	-0.0117	-0.0069	-0.0172	0.0033	-0.0093	-0.0006
**CD40**	1.6639	1.2526	1.7019	1.3159	-0.0211	0.0090	-0.0163	-0.0045	-0.0164	0.0042	0.0092	-0.0041
**ADORA2A**	0.0857	0.0805	0.0930	0.0565	-0.0063	0.0155	0.0078	-0.0151	-0.0056	0.0015	0.0026	-0.0013
**TLR4**	0.7458	0.9877	1.0592	0.7817	-0.0059	0.0081	0.0002	-0.0072	-0.0035	0.0035	-0.0022	-0.0027
**HAVCR2**	1.1448	1.1057	1.3836	0.8393	-0.0187	0.0038	-0.0315	0.0023	-0.0098	0.0057	0.0051	-0.0054
**ITGB2**	1.7275	1.5556	1.8711	1.3055	-0.0146	0.0081	-0.0066	-0.0056	-0.0100	0.0034	0.0031	-0.0029
**ICAM1**	1.8762	1.5580	2.0402	1.4881	-0.0173	0.0107	-0.0060	-0.0079	-0.0084	0.0069	-0.0058	-0.0050
**SELP**	0.5683	0.5351	0.6488	0.5996	-0.0233	0.0112	-0.0026	-0.0084	0.0033	0.0017	0.0011	-0.0020
**HLA-DRB5**	2.6365	2.1681	2.5815	2.1948	0.0102	0.0005	-0.0005	-0.0011	-0.0183	0.0077	0.0176	-0.0084
**HLA-DQA1**	1.8627	1.4800	1.9484	1.3870	0.0102	0.0005	-0.0005	-0.0011	-0.0183	0.0077	0.0176	-0.0084
**HLA-DQB1**	2.2295	1.6782	2.0907	1.6972	0.0102	0.0005	-0.0005	-0.0011	-0.0183	0.0077	0.0176	-0.0084
**MICA**	1.3878	1.3270	1.3968	1.3667	0.0102	0.0005	-0.0005	-0.0011	-0.0183	0.0077	0.0176	-0.0084
**MICB**	1.0693	0.8610	1.2010	0.7497	0.0102	0.0005	-0.0005	-0.0011	-0.0183	0.0077	0.0176	-0.0084
**HLA-DQA2**	1.7196	1.2854	1.7498	1.2424	0.0102	0.0005	-0.0005	-0.0011	-0.0183	0.0077	0.0176	-0.0084
**HLA-**	1.6699	1.0870	1.5824	1.1016	0.0102	0.0005	-0.0005	-0.0011	-	0.0077	0.0176	-

**DQB2**									0.0183			0.0084
**HLA-B**	3.5472	2.9744	3.3752	3.2447	0.0102	0.0005	-0.0005	-0.0011	-0.0183	0.0077	0.0176	-0.0084
**HLA-A**	3.4341	2.8875	3.2356	3.1685	0.0072	0.0025	-0.0100	-0.0014	-0.0112	0.0079	0.0227	-0.0099
**HLA-C**	3.3619	2.9029	3.2004	3.1143	0.0102	0.0005	-0.0005	-0.0011	-0.0183	0.0077	0.0176	-0.0084
**HLA-DRA**	3.3900	3.0198	3.4367	2.9671	0.0102	0.0005	-0.0005	-0.0011	-0.0183	0.0077	0.0176	-0.0084
**HLA-DRB1**	3.1861	2.7301	3.1601	2.7442	0.0102	0.0005	-0.0005	-0.0011	-0.0183	0.0077	0.0176	-0.0084
**HLA-DPB1**	2.6109	2.1965	2.5544	2.1987	0.0116	0.0002	-0.0107	0.0005	-0.0183	0.0077	0.0125	-0.0076
**HLA-DPA1**	2.3150	1.9591	2.3219	1.9226	0.0116	0.0002	-0.0107	0.0005	-0.0185	0.0080	0.0122	-0.0079
**IDO1**	1.3350	0.7901	1.5796	0.7838	-0.0385	0.0247	0.0358	-0.0255	-0.0173	0.0044	-0.0076	-0.0018
**GZMA**	1.6443	1.0393	1.6682	1.0802	-0.0074	0.0042	0.0024	-0.0037	-0.0207	0.0057	-0.0099	-0.0024
**PRF1**	1.3762	0.8006	1.3650	0.8106	-0.0198	0.0082	0.0057	-0.0071	-0.0067	0.0019	0.0014	-0.0015
**ARG1**	0.0855	0.1031	0.0873	0.0949	0.0000	0.0094	0.0007	-0.0088	-0.0227	0.0055	-0.0094	-0.0021
**HMGB1**	1.9774	1.9661	1.9417	1.8891	-0.0168	0.0104	0.0012	-0.0086	-0.0147	-0.0024	0.0111	0.0016
**ENTPD1**	0.9890	1.0906	1.1167	0.9566	-0.0131	0.0054	0.0040	-0.0047	-0.0085	0.0038	0.0071	-0.0040

Pan-cancer samples are divided into four groups based on PD-L1 expression and TIL Z score as follows: type I, PD-L1 positive with TIL positive; type II, PD-L1 negative with TIL negative; type III, PD-L1 positive with TIL negative; and type IV, PD-L1 negative with TIL positive. Amplification frequency (the difference between the fraction of samples in which an immune gene is amplified in a particular subtype and the amplification fraction in all samples); and the deletion frequency (as amplifications) for 75 immune genes by immune subtype. Abbreviations: log10(TPM median + 1), the log10 value of the median of TPM plus one.

Table S9. The pathway scores of the shared pathways across the four subtypes of tumor immune microenvironment.

Pathway	Pathway score			
	Type I	Type II	Type III	Type IV
**Complement and coagulation cascades**	0.3925	0.0856	0.3512	0.1707
**Retinol metabolism**	0.3493	0.0733	0.3868	0.1906
**Drug metabolism - cytochrome P450**	0.3646	0.0661	0.3819	0.1874
**Metabolism of xenobiotics by cytochrome P450**	0.3763	0.0613	0.3882	0.1742
**Chemical carcinogenesis**	0.3823	0.0681	0.3812	0.1684
**Steroid hormone biosynthesis**	0.3608	0.0637	0.4422	0.1332
**Drug metabolism - other enzymes**	0.3890	0.0582	0.4255	0.1274
**Ascorbate and aldarate metabolism**	0.3562	0.0846	0.4158	0.1434
**Glucuronate pathway**	0.4071	0.0593	0.4037	0.1299
**Pentose and glucuronate interconversions**	0.4071	0.0593	0.4037	0.1299
**Porphyrin and chlorophyll metabolism**	0.4071	0.0593	0.4037	0.1299
**Fat digestion and absorption**	0.3525	0.0435	0.4726	0.1314
**Vitamin digestion and absorption**	0.3877	0.0480	0.4859	0.0784
**Bile secretion**	0.4251	0.1051	0.3327	0.1370
**Pancreatic secretion**	0.3708	0.1056	0.4237	0.0999
**Protein digestion and absorption**	0.3598	0.1167	0.4335	0.0901
**Tyrosine metabolism**	0.2231	0.0611	0.5195	0.1964
**Glycolysis / Gluconeogenesis**	0.3381	0.0856	0.4206	0.1557
**Fatty acid degradation**	0.3016	0.1124	0.4034	0.1825
**Primary bile acid biosynthesis**	0.3489	0.1102	0.3517	0.1891
**PPAR signaling pathway**	0.4308	0.0769	0.3531	0.1392
**Bile acid biosynthesis, cholesterol**	0.3480	0.1046	0.3550	0.1924
**Linoleic acid metabolism**	0.2511	0.0965	0.5003	0.1521
**Arachidonic acid metabolism**	0.2808	0.0708	0.5192	0.1291

Pan-cancer samples are divided into four groups based on PD-L1 expression and TIL Z score as follows: type I, PD-L1 positive with TIL positive; type II, PD-L1 negative with TIL negative; type III, PD-L1 positive with TIL negative; and type IV, PD-L1 negative with TIL positive.

Table S10. Statistical data of the specific gene TPM across the four subtypes of tumor immune microenvironment.

Genes	x±σ				
	**Type I**	**Type II**	**Type III**	**Type IV**	**p value**
**IFNG**	3.6821±4.9707	0.9872±2.8601	6.2627±12.3615	0.9642±2.0909	<2.2e-16
**TNF**	5.5755±12.5646	5.4704±11.8647	8.8042±20.2282	3.2863±8.2091	<2.2e-16
**IL12A**	1.3549±3.1969	1.8349±3.391	1.8474±2.7986	1.0006±2.2759	<2.2e-16
**IL12B**	0.6232±1.457	0.4756±4.2685	1.1769±6.6174	0.2447±0.904	<2.2e-16
**VEGFA**	47.1882±75.8538	59.906±112.5433	55.4688±60.8448	68.5698±118.2574	1.2e-07
**TGFB1**	94.7305±54.7422	85.5343±71.2495	118.3545±83.4194	64.2295±60.054	<2.2e-16
**IL6**	13.8718±93.1519	9.7069±34.4149	23.8079±50.4288	5.3844±17.4216	<2.2e-16
**IL10**	1.7147±2.772	1.8251±8.8754	4.2433±15.7911	0.8615±1.6081	<2.2e-16
**GZMB**	40.2642±49.1587	12.0498±25.8512	57.189±96.4566	13.1238±53.808	<2.2e-16
**PRF1**	29.1039±28.8787	10.8678±19.7757	38.2342±53.6238	11.1715±17.645	<2.2e-16

Pan-cancer samples are divided into four groups based on PD-L1 expression and TIL Z score as follows: type I, PD-L1 positive with TIL positive; type II, PD-L1 negative with TIL negative; type III, PD-L1 positive with TIL negative; and type IV, PD-L1 negative with TIL positive.

Abbreviations: x±σ, mean ± standard deviation; IFNG, interferon gamma; TNF, tumor necrosis factor; IL12A, interleukin 12A; IL12B, interleukin 12B; VEGFA, vascular endothelial growth factor A; TGFB1, transforming growth factor beta 1; IL6, interleukin 6; IL10, interleukin 10; GZMB, granzyme B; PRF1, perforin-1.

Table S11. Statistical data of 8 immune cell proportion across the four subtypes of tumor immune microenvironment in validation cohort from the Gene Expression Omnibus database.

**Immune cell types**	**x±σ**				
	**Type I**	**Type II**	**Type III**	**Type IV**	**p value**
**T cells**	0.4328±0.0731	0.2641±0.0847	0.3544±0.0943	0.3294±0.0983	<2.2e-16
**B cells**	0.0429±0.0349	0.0194±0.0226	0.0237±0.0335	0.0258±0.0283	<2.2e-16
**Macrophages**	0.4095±0.0957	0.547±0.1239	0.5032±0.1145	0.4897±0.1215	<2.2e-16
**DC cells**	0.0105±0.0168	0.0101±0.0154	0.0117±0.0201	0.0111±0.0165	0.31
**NK cells**	0.0596±0.0251	0.0387±0.0223	0.0493±0.0274	0.0459±0.0228	<2.2e-16
**Mast cells**	0.0419±0.0286	0.1179±0.0726	0.0546±0.0521	0.0966±0.0619	<2.2e-16
**Eosinophils**	0.0±0.0	0.0±0.0006	0.0±0.0	0.0±0.0005	0.6
**Neutrophils**	0.0028±0.0153	0.0029±0.0094	0.003±0.0074	0.0015±0.0043	1.9e-05

Breast cancer samples are divided into four groups based on PD-L1 expression and TIL Z score as follows: type I, PD-L1 positive with TIL positive; type II, PD-L1 negative with TIL negative; type III, PD-L1 positive with TIL negative; and type IV, PD-L1 negative with TIL positive. Abbreviations: x±σ, mean ± standard deviation; DC cells, dendritic cells; NK cells, Natural killer cells.

Table S12. The relative proportion of 20 Immune cell subtypes across the four subtypes of tumor immune microenvironment in validation cohort from the Gene Expression Omnibus database.

Immune cell subtypes	Relative proportion			
	Type I	Type II	Type III	Type IV
**T cells CD8**	37.8199	29.39244	34.82628	31.9061
**T cells CD4 naive**	0	0.005558	0	0.000943
**T cells CD4 memory resting**	43.69211	57.3395	48.31294	55.24973
**T cells CD4 memory activated**	2.000126	0.071391	1.097853	0.237152
**T cells follicular helper**	7.59751	7.240071	7.87873	5.852387
**T cells gamma delta**	0.448493	0.073389	0.466406	0.11765
**T cells regulatory (Tregs)**	8.441861	5.87765	7.417789	6.636037
**B cells naive**	74.06252	74.56335	72.51838	76.04757
**B cells memory**	23.2216	16.94169	20.77287	16.53687
**Plasma cells**	2.715881	8.494962	6.708746	7.41556
**Dendritic cells resting**	79.86451	90.80358	78.48169	90.61175
**Dendritic cells activated**	20.13549	9.196421	21.51831	9.388251
**NK cells resting**	0.085976	5.476928	0.98342	1.449035
**NK cells activated**	99.91402	94.52307	99.01658	98.55096
**Mast cells resting**	97.19886	93.85222	92.07631	95.20349
**Mast cells activated**	2.801139	6.147775	7.92369	4.796508
**Monocytes**	5.567441	5.912497	6.076638	6.1949
**Macrophages M0**	21.96947	20.33371	24.84728	17.62694
**Macrophages M1**	24.55563	9.010947	16.52947	13.72083
**Macrophages M2**	47.90746	64.74284	52.5466	62.45733

Breast cancer samples are divided into four groups based on PD-L1 expression and TIL Z score as follows: type I, PD-L1 positive with TIL positive; type II, PD-L1 negative with TIL negative; type III, PD-L1 positive with TIL negative; and type IV, PD-L1 negative with TIL positive. The relative proportion refers to the proportion of each immune cell subtype to the immune cell type which it belongs.

Table S13. Statistical data of the specific gene TPM across the four subtypes of tumor immune microenvironment in validation cohort from the Gene Expression Omnibus database.

Genes	x±σ				
	Type I	Type II	Type III	Type IV	p value
IFNG	1.3761±1.1004	0.172±0.1604	1.197±1.2829	0.155±0.1951	<2.2e-16
TNF	2.0154±2.2279	0.779±1.0797	2.2545±2.9247	0.5041±0.6719	<2.2e-16
IL12A	0.4873±0.6421	0.272±0.3575	0.6166±0.5665	0.1731±0.2657	<2.2e-16
IL12B	0.3923±0.296	0.1322±0.0892	0.3798±0.3122	0.0976±0.0867	<2.2e-16
VEGFA	23.6823±37.3677	23.1697±20.3401	37.5709±32.1312	10.9173±12.1465	<2.2e-16
TGFB1	35.7903±15.8189	36.8513±22.7435	57.1662±29.7121	20.2818±13.3514	<2.2e-16
IL6	2.2228±6.7072	1.515±2.9958	4.6579±12.2949	0.8278±2.169	<2.2e-16
IL10	0.8486±0.47	0.4593±0.3801	1.204±1.4405	0.2791±0.2449	<2.2e-16
GZMB	19.6877±12.8835	1.8425±3.0091	17.652±16.3429	2.2164±3.7294	<2.2e-16
PRF1	8.5772±5.4426	1.3393±1.1831	7.9223±6.4884	1.2897±1.3609	<2.2e-16

Breast cancer samples are divided into four groups based on PD-L1 expression and TIL Z score as follows: type I, PD-L1 positive with TIL positive; type II, PD-L1 negative with TIL negative; type III, PD-L1 positive with TIL negative; and type IV, PD-L1 negative with TIL positive.

Abbreviations: x±σ, mean ± standard deviation; IFNG, interferon gamma; TNF, tumor necrosis factor; IL12A, interleukin 12A; IL12B, interleukin 12B; VEGFA, vascular endothelial growth factor A; TGFB1, transforming growth factor beta 1; IL6, interleukin 6; IL10, interleukin 10; GZMB, granzyme B; PRF1, perforin-1.
