# Supplementary figures and images for: Multi-Omics Perspective Reveals the Different Patterns of Tumor Immune Microenvironment Based on Programmed Death Ligand 1 (PD-L1) Expression and Predictor of Responses to Immune Checkpoint Blockade across Pan-Cancer
Source: Int J Mol Sci. 2021 May 13;22(10):5158. doi: 10.3390/ijms22105158 (PMC8153013; doi:10.3390/ijms22105158)

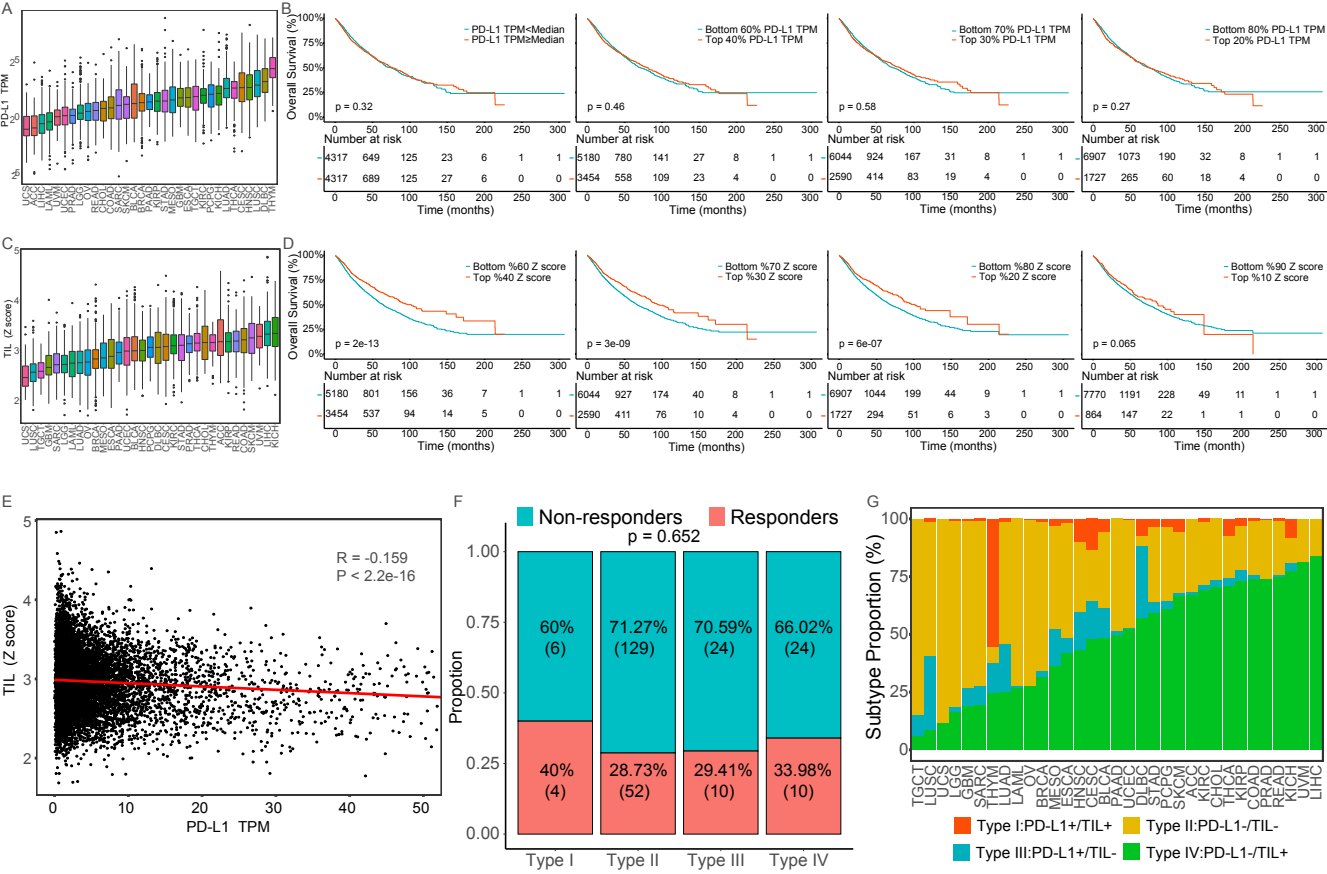

Supplement: Supplementary file 1 [file ijms-22-05158-s001.zip › Supplymentary Figure1.pdf]

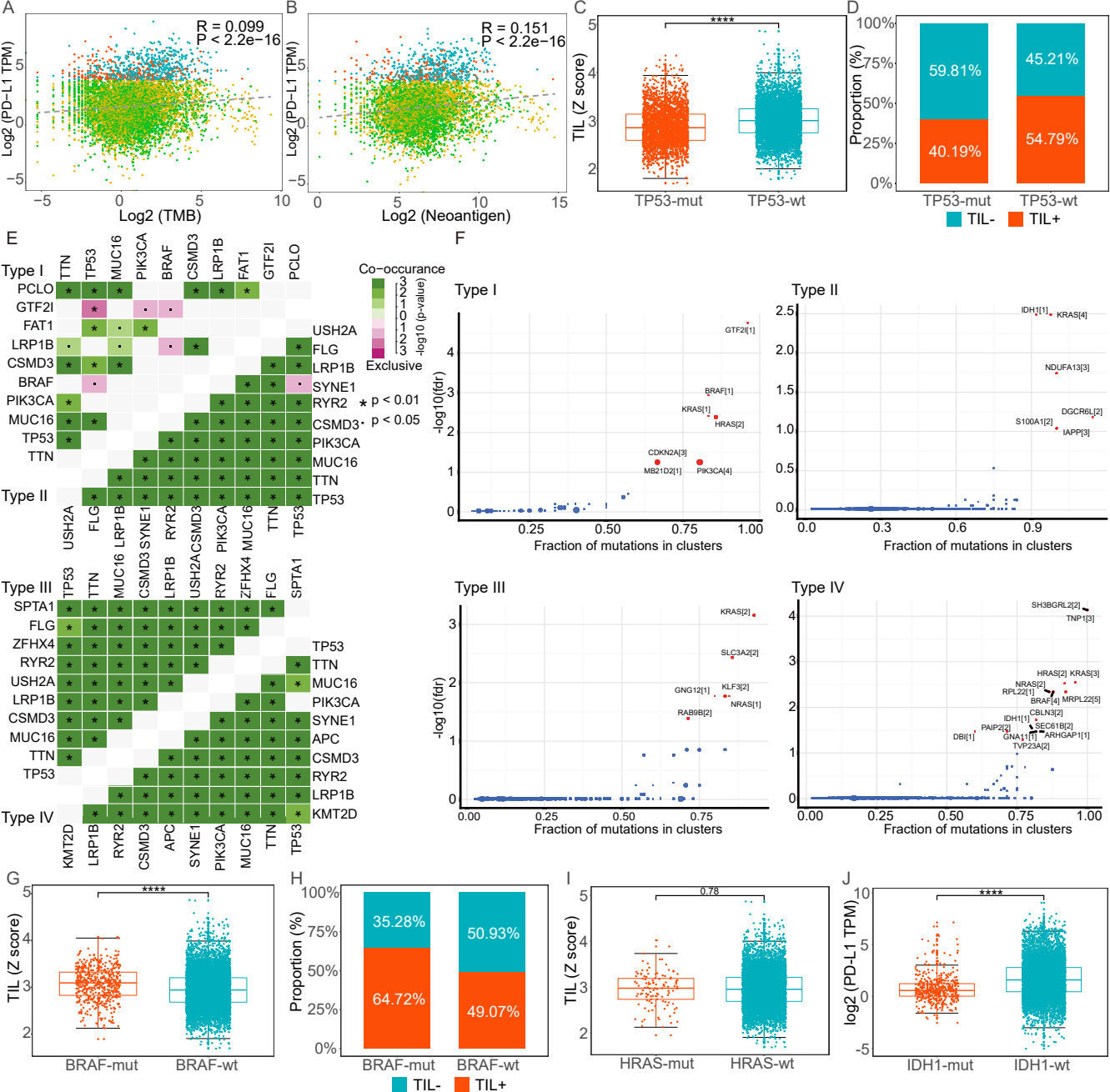

Supplement: Supplementary file 1 [file ijms-22-05158-s001.zip › Supplymentary Figure2.pdf]

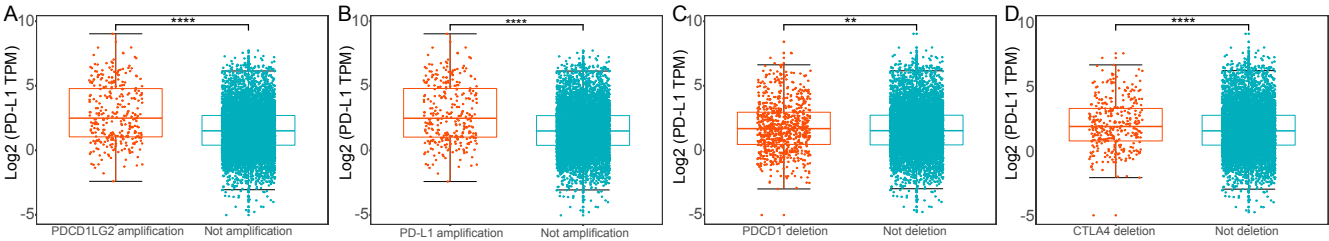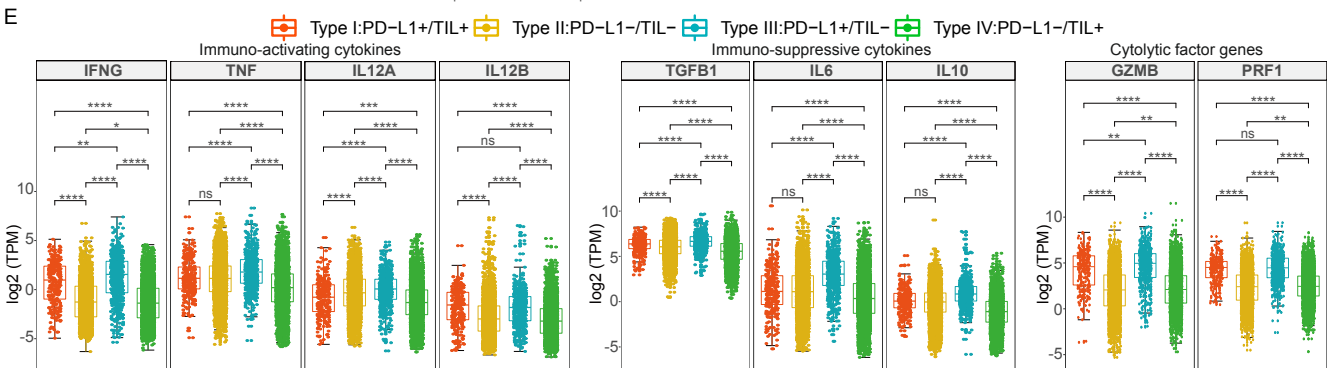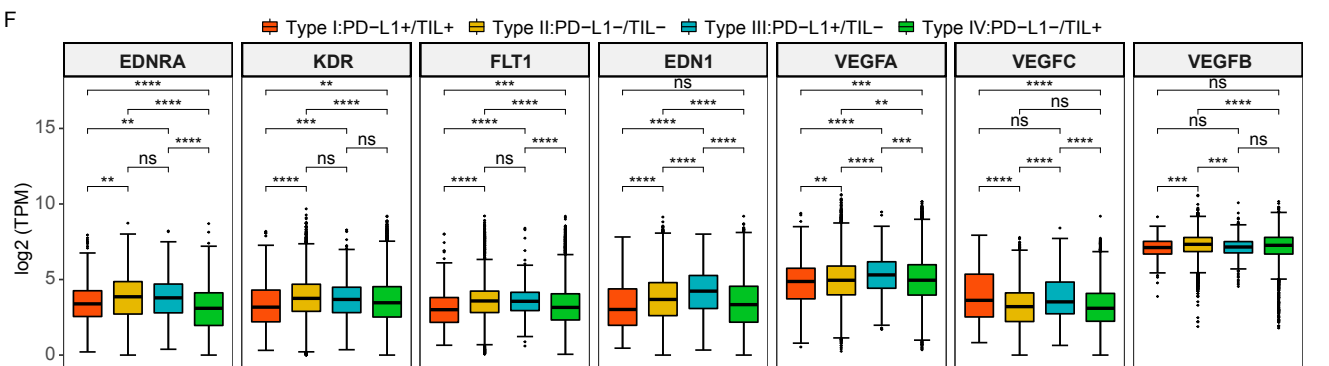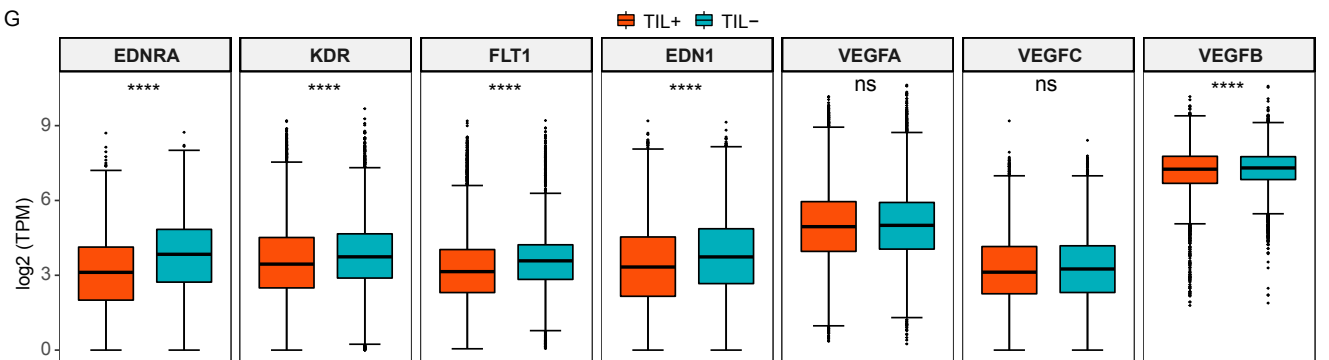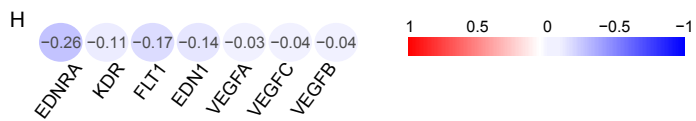

Supplement: Supplementary file 1 [file ijms-22-05158-s001.zip › Supplymentary Figure3.pdf]

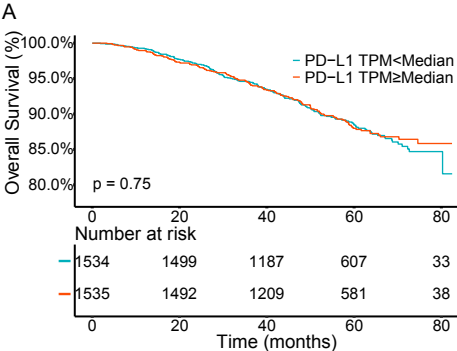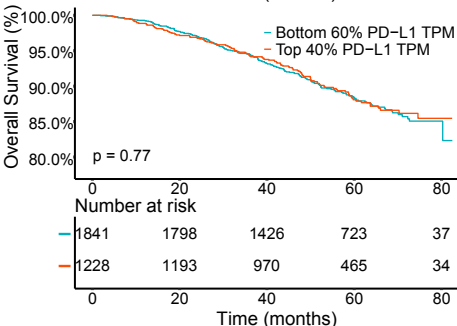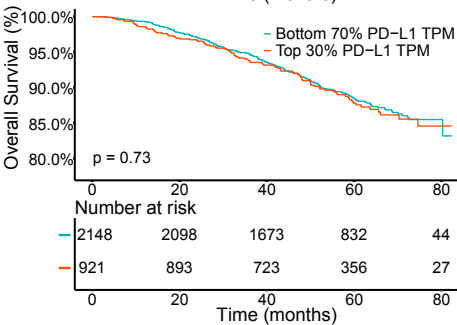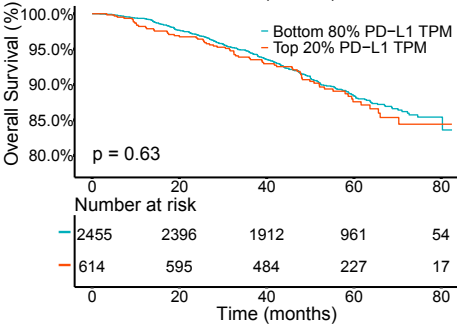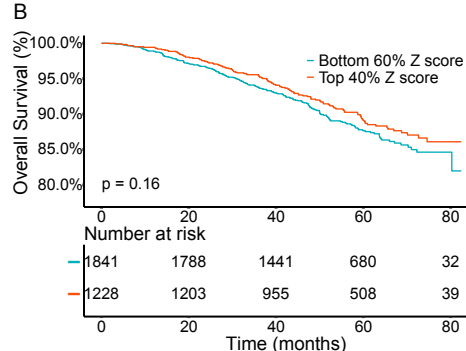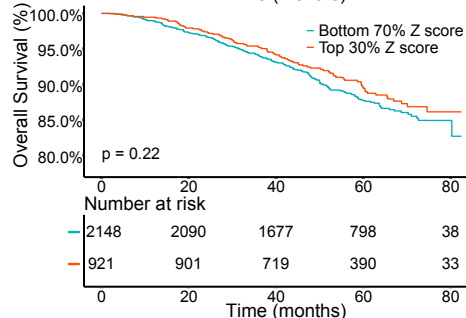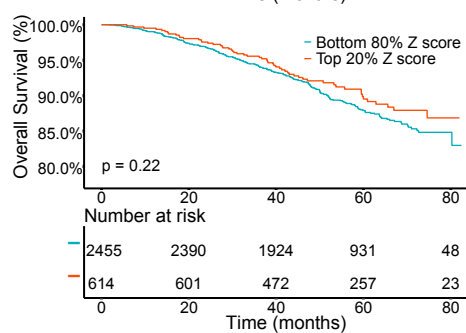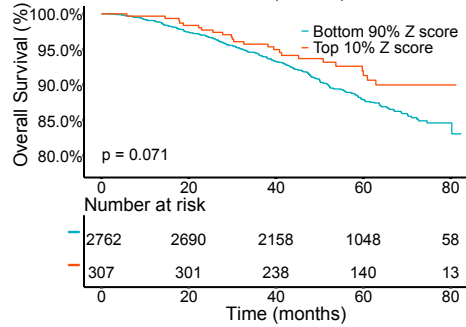

Supplement: Supplementary file 1 [file ijms-22-05158-s001.zip › Supplymentary Figure4.pdf]

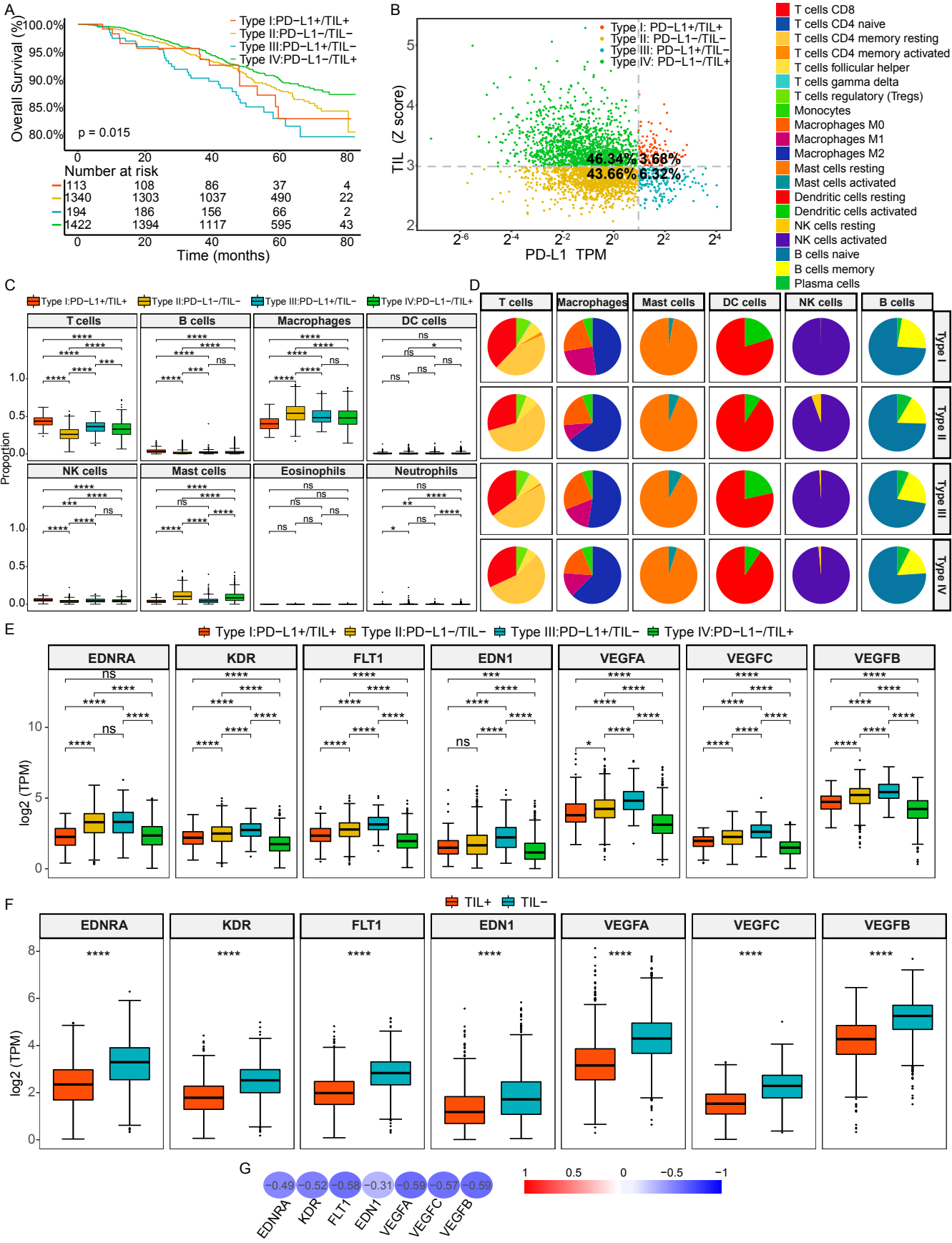

Supplement: Supplementary file 1 [file ijms-22-05158-s001.zip › Supplymentary Figure5.pdf]
